# A native, highly active *Tc1/mariner* transposon from zebrafish (*ZB*) offers an efficient genetic manipulation tool for vertebrates

**DOI:** 10.1093/nar/gkab045

**Published:** 2021-02-08

**Authors:** Dan Shen, Chengyi Song, Csaba Miskey, Shuheng Chan, Zhongxia Guan, Yatong Sang, Yali Wang, Cai Chen, Xiaoyan Wang, Ferenc Müller, Zoltán Ivics, Bo Gao

**Affiliations:** College of Animal Science & Technology, Yangzhou University, Yangzhou, Jiangsu 225009, China; Division of Medical Biotechnology, Paul Ehrlich Institute, Langen 63225, Germany; College of Animal Science & Technology, Yangzhou University, Yangzhou, Jiangsu 225009, China; Division of Medical Biotechnology, Paul Ehrlich Institute, Langen 63225, Germany; College of Animal Science & Technology, Yangzhou University, Yangzhou, Jiangsu 225009, China; College of Animal Science & Technology, Yangzhou University, Yangzhou, Jiangsu 225009, China; College of Animal Science & Technology, Yangzhou University, Yangzhou, Jiangsu 225009, China; College of Animal Science & Technology, Yangzhou University, Yangzhou, Jiangsu 225009, China; College of Animal Science & Technology, Yangzhou University, Yangzhou, Jiangsu 225009, China; College of Animal Science & Technology, Yangzhou University, Yangzhou, Jiangsu 225009, China; Institute of Cancer and Genomic Sciences, Birmingham Centre for Genome Biology, College of Medical and Dental Sciences, University of Birmingham, Edgbaston, Birmingham B15 2TT, UK; Division of Medical Biotechnology, Paul Ehrlich Institute, Langen 63225, Germany; College of Animal Science & Technology, Yangzhou University, Yangzhou, Jiangsu 225009, China

## Abstract

New genetic tools and strategies are currently under development to facilitate functional genomics analyses. Here, we describe an active member of the *Tc1/mariner* transposon superfamily, named *ZB*, which invaded the zebrafish genome very recently. *ZB* exhibits high activity in vertebrate cells, in the range of those of the widely used transposons *piggyBac* (*PB*), *Sleeping Beauty* (*SB*) and *Tol2*. *ZB* has a similar structural organization and target site sequence preference to *SB*, but a different integration profile with respect to genome-wide preference among mammalian functional annotation features. Namely, *ZB* displays a preference for integration into transcriptional regulatory regions of genes. Accordingly, we demonstrate the utility of *ZB* for enhancer trapping in zebrafish embryos and in the mouse germline. These results indicate that *ZB* may be a powerful tool for genetic manipulation in vertebrate model species.

## INTRODUCTION

DNA transposons, a class of genetic elements that can ‘jump’ to different locations within a genome, were first described by Barbara McClintock while working on maize (1) and are widespread across prokaryotic and eukaryotic organisms (2,3). The transposition of DNA transposons does not involve an RNA intermediate, as is the case for retrotransposons; rather, they are represented by the classic cut-and-paste, rolling-circle-like replication (Helitrons) or self-replication (Polintons, alternatively known as Mavericks) mechanisms (4,5). The cut-and-paste DNA transposons could be classified into 17 superfamilies, i.e. *Tc1/mariner*, *Zator*, *Merlin*, *PIF/Harbinger*, *MULE* (*Mutator*-like element), *P*, *hAT* (hobo/Ac/Tam3), *Kolobok*, *Novosib*, *piggyBac*, *Sola1*, *Sola2*, *Sola3*, CMC (*CACTA*, *Mirage* and *Chapaev*), *Transib*, *Academ* and *Ginger*, based on the alignment of the catalytic domain of transposase, which is an acidic amino acid triad (DDE or DDD) that catalyses the cut-and-paste transposition reaction (6). Cut-and-paste DNA transposons are mobilized by their respective transposases in *trans*, and are thus suitable for developing genetic tools for versatile gene-delivery and gene-discovery applications, ranging from transgenesis to functional genomics and gene therapy (7,8). In particular, transposons can be applied as vectors for germline transgenesis, and as insertional mutagens. The major advantage of using cut-and-paste transposons as mutagenesis tools is that they facilitate the analysis of functional genomic elements (such as enhancers), gene function and activity in an easy, controlled and scalable manner (9,10). *ISY100*, *Tn5*, *Tn10* and *Mu* represent a common choice for the mutagenesis of prokaryotic genomes (11–13), while at least 10 transposons, including *P* element, *Mos1*, *Minos*, *Sleeping Beauty (SB)*, *piggyBac (PB)* and *Tol2*, have been exploited for gene-transfer applications in eukaryotes. In particular, *SB*, *PB* and *Tol2* have been successfully used in vertebrate models, including zebrafish, frog and mice, for transgenesis and mutagenesis (14,15). *Tol2*, which belongs to the superfamily *hAT*, was the first active autonomous transposon isolated in vertebrate species (16). Various other active members of this group, including *Ac/Ds* (1), *Hobo* (17), *Hermes* (18), *TcBuster* (19) and *Tgf2* (20), have been isolated or developed in eukaryotes. However, only *Tol2* has been widely used as a genetic tool, particularly in the zebrafish model (21,22). The *PB* transposon is a member of the *piggyBac* transposon superfamily present in the genomes of a wide range of organisms, including fungi, plants, insects, crustaceans, urochordates, amphibians, fishes and mammals (23,24). In addition, putatively active *PB*-like transposons have been identified in moths (25), silkworms (26), ants (27), Xenopus (28) and bats (29). *SB* (30), *Passport* (31), *Frog Prince* (32) and *Hsmar1* (33) are elements of the *Tc1/mariner* superfamily that have been revived and reconstructed from inactive elements based on phylogenetic analyses. This group may represent the most widespread DNA transposons in nature, and 14 individual elements (such as *Minos*, *Mos1*, *Fot1* and *Impala*) are active in their natural forms (14,31,34–36). However, only the synthetic *SB* transposon exhibits high activity and displays great potential as a genetic tool in vertebrates (7,8,15).

The integration site preferences of particular transposons, such as *PB*, *SB* and *Tol2*, are substantially different and can greatly affect the utility of transposon vectors for different applications. Furthermore, the insertional biases associated with a specific vector system also represent a main limitation to full genome coverage using individual transposon-based vectors (8). To expand the range of tools available for the genetic manipulation of different organisms, we describe and functionally characterize *ZB*, a member of the *Tc1/mariner* superfamily, which likely represents the most active *Tc1/mariner* transposon in the zebrafish genome. We demonstrate that *ZB* is capable of robust transposition in vertebrate cells, which can be translated to highly efficient mutagenesis and enhancer trapping (ET) screening in zebrafish and mice. *ZB* therefore represents an alternative, powerful tool for transgenesis and mutagenesis in vertebrates and possibly a new gene therapy delivery system for humans.

## MATERIALS AND METHODS

### Animals

The Tubingen strain of zebrafish (*Danio rerio*) was purchased from the China Zebrafish Resource Centre. Wild-type (WT) FVB mice were purchased from Animal Experimental Centre of Yangzhou University. All treatments and protocols involving zebrafish and mouse used in this study were strictly carried out in accordance with the guidelines of the Animal Experiment Ethics Committee of Yangzhou University.

### TE annotation in zebrafish

The zebrafish (*D. rerio*, GRCz11) genome was downloaded from the NCBI genome database. The genome coverage of *Tc1/mariner* transposons in the zebrafish genome was annotated using the RepeatMasker program (http://www.repeatmasker.org/) with custom zebrafish-specific repeat libraries, which combine known repeats from Repbase (https://www.girinst.org/repbase/) and the new elements identified by the TBlastN, MITE-Hunter and RepeatModeler (http://repeatmasker.org/RepeatModeler/) programs. All identified *Tc1/mariner* transposons in the zebrafish genome are provided in Supplemental Dataset 1. The phylogenetic trees of autonomous *Tc1/mariner* transposons were inferred using the maximum likelihood method within the IQ-TREE program (37), based on the alignment of the conserved catalytic ‘DDE/D’ domain with an ultrafast bootstrap value of 1000. The best model was selected using ModelFinder embedded in the IQ-TREE program (37), and multiple alignments were performed using the MAFFT program (38). Reference elements of the *Tc1/mariner* families were downloaded from GenBank. Protein secondary structure predictions were performed using the PSIPRED program (http://bioinf.cs.ucl.ac.uk/psipred/). NLS predictions were performed using the PSORT program (http://psort.hgc.jp/) and PROSITE (http://prosite.expasy.org/). The insertion time of each element was estimated using the Kimura two-parameter distance (*K*) with the formula: *t* = *K*/2*r* (39). The Kimura two-parameter distance was computed using the calcDivergenceFromAlign.pl package from RepeatMasker (40). An average substitution rate (*r*) of 4.13 **×** 10^−9^ for substitutions per synonymous site per year was applied (41). Based on the structure organization and insertion age analysis, the youngest transposon (Tc1–8B_DR, named as *ZB*), displaying multiple intact copies in the genome and with high current activity, was selected for further study.

### Plasmids for mammalian cells

A two-plasmid transposition assay, as described previously (42), including a donor plasmid containing a transposon carrying a neomycin resistance cassette and a helper plasmid expressing the transposase, was applied to test transposition activity in cells. The *ZB* transposase expression vector, as a helper plasmid (pCZBNpA), was created by replacing the SB100X-transposase-coding sequence with *ZB* in the pCSBNpA backbone using the *XhoI/NotI* enzymes (42). pUC19SBneo containing a neo expression cassette was used to create the donor plasmid of *ZB* by replacing the *SB* terminal inverted repeats (TIRs) with *ZB* TIRs. The remaining three transposase expression vectors (pCSBNpA, pCmPBNpA and pCTol2NpA) and donor vectors (pUC19SBneo, pUC19PBneo and pUC19Tol2neo) were the same as used in Grabundzija et al. (42). The *ZB* transposase coding region and TIRs were cloned by PCR from the zebrafish genome using the primers listed in Supplementary Table S1, followed by verification by sequencing (TsingKe, China). We selected those *ZB* 5’ and 3’ TIR, and transposase TA clones (relevant sequences are listed in Supplementary Table S2), which represent relatively high sequence identity to the consensus.

### Plasmids for ET in zebrafish and mice

To prepare the backbone of pZB-Msc, the cloned *ZB* TIRs were subcloned into the *Asc1/Msc1* and *Nru1/Fse1* site of pT2-HB (#26557; Addgene, USA). Subsequently, the fragment containing the *Krt4* mini-promoter, the *GFP* reporter and the polyA from pTol2-Krt4-GFP (43) was cloned into the Msc1 site of pZB-Msc to produce pZB-Krt4-GFP, which was used for ET in zebrafish. The *Krt4* mini-promoter of pZB-Krt4-GFP was replaced with the *Myc* promoter from the mouse genome with *SpeI/EcoRI* restriction sites to obtain pZB-Myc-GFP for ET in mice. To produce pSB-PGK2-ZBase, a fragment containing the testis-specific human *PGK2* promoter (44), IRES, the *ZB* transposase coding sequence, rabbit globin polyA and the SV40 enhancer was cloned into the *Xma1/Pac1* sites of pT2-HB. The *ZB* transposase coding region was also subcloned into the pTNT^TM^ vector (AL5610; Promega, USA), to produce pTNT-ZB, which was used as a template to synthesize the *ZB* transposase mRNA. All primers used in this experiment are listed in Supplementary Table S1.

### Transposition assay

HepG2 cells were maintained in Dulbecco's modified Eagle's medium (Gibco, USA) supplemented with 10% foetal bovine serum (Gibco, USA) and 1% P/S (Gibco, USA). Cells (3 × 10^5^) were seeded onto each well of six-well plates 1 day prior to transfection. The cells were transfected with 1.5 μg of DNA consisting of the donor plasmid (500 or 10 ng) and increasing amounts of helper plasmids (from 0 to 1000 ng) using 3 μl of Trans1T-LT1 Reagent (Mirus, USA). We conducted three replicates per concentration. Twenty-four hours post-transfection, cells were replated onto 10 cm plates (10% of the transfected cells from each well were replated for HepG2 cells, whereas 1% we replated for HeLa cells) and selected in 1 mg/ml G418 medium. After 2 weeks of selection, the resistant colonies were stained with methylene blue and analysed by ImageJ (https://imagej.net/Welcome).

### Footprint analysis

The genomic DNA was isolated from HepG2 cells transfected with pUC19ZBneo and pCZBNpA at 2 days post-transfection using a tissue and blood DNA extraction kit(Qiagen, Germany). Primers flanking the transposon were used to amplify PCR products, to identify the footprints left behind by *ZB* transposition in the donor plasmids via sequencing. About 2 μg of template DNA was used for PCR with the pUC19-backbone-specific primers puc1F/R, puc2F/R and puc3F/R. The PCR products were cloned into the pJET1.2 vector (Thermo Fisher, USA) and sequenced (TsingKe, China).

### Insertion site library preparation and bioinformatics analysis

The preparation of the Illumina sequencing-compatible insertion site libraries was described earlier (45). Briefly, genomic DNA was extracted from HepG2 G418 resistant colonies using a DNeasy Blood and Tissue Kit (Qiagen, Germany). DNA samples were sonicated to an average length of 600 bp using a Covaris M220 ultrasonicator (Covaris, USA). Fragmented DNA was subjected to end repair, dA-tailing and linker ligation steps. Transposon-genome junctions were then amplified by nested PCRs using two primer pairs binding to the transposon TIR and the linker, respectively. The PCR products were separated on a 1.5% ultrapure agarose gel and a size range of 200–500 bp was extracted from the gel. Some of the generated product was cloned and Sanger sequenced for library verification before high-throughput sequencing with a NextSeq (Illumina) instrument with single-end 150 bp setting. Then, essentially, nested primers (ZBnest1, ZBnest2, LinkerNest1 and LinkerNest2) were used to perform nested PCR (listed in Supplementary Table S1). The conditions and thresholds of the raw read processing and mapping parameters have been specified previously (45). In short, the raw reads were subjected to quality trimming, and the resulting reads were mapped to the hg38 human genome assembly with *bowtie* (46) in cycling mapping using the TAPDANCE algorithm (47).

The coordinates of genic features, histone modification-related Chip-Seq peak regions, HapG2-specific chromatin segmentation data and open chromatin datasets were downloaded from the UCSC Table Browser (https://genome.ucsc.edu/cgi-bin/hgTables) for the hg38 genome assembly. Insertion site frequencies of *ZB* and *SB* within these regions above were compared to a set of 100 000 computationally generated random loci in the human genome.

### Generation and analysis of transgenic zebrafish

The amounts of transposase mRNA and transposon donor plasmid of both the *ZB* and *Tol2* systems for microinjection in zebrafish embryos were optimized as described previously (43). Subsequently, ∼1 nl of DNA and RNA mixtures containing 20 ng/μl of circular donor plasmid (pZB-Krt4-GFP or pTol2-Krt4-GFP) and 30 ng/μL of the transposase mRNA were injected into fertilized eggs for the generation of transgenic zebrafish. After injection, *GFP* expression was screened at 1 and 5 days postfertilization(dpf) by fluorescence microscopy. GFP-positive embryos at 5 dpf were raised to adulthood and crossed at least five times to wild-type (WT) fish for *GFP* germline transfer analysis, and the *GFP* expression of F1 embryos was screened at 1 and 5 dpf under a Leica M165FC fluorescence microscope (Solms, Germany). Zebrafish were maintained at 28.5°C in a licensed aquarium facility (ESEN, China), according to standard protocols. *GFP* reporter expression patterns were analysed for tissue specificity by fluorescence microscopy at 3 stages of development (12 hpf, 2 dpf and 3 dpf) and compared with expression pattern (whole-mount RNA in situ hybridization) of genes residing in the environment of identified integration sites. Expression patterns of flanking genes residing within 400 kb were retrieved from zfin.org (http://zfin.org/).

### Generation of transgenic mice

The transposon vectors, termed pZB-Myc-GFP and pT2-PGK2-ZBase, were purified from the Endofree plasmid kit (Qiagen, Germany). ZBase- and SBase-mRNA were prepared in vitro using an mMESSAGE mMACHINE T7 Kit (Ambion, USA), according to the manufacturer's instructions. The DNA was resuspended in injection TE buffer (Invitrogen, USA) at a concentration of 20 ng/μl and mixed with the corresponding transposase mRNA at a concentration of 50 ng/μl. Pronuclear injections were performed according to a well-established protocol (48) and FVB mice were used throughout the study. Because the offsprings of *nl11* and *nl9* mice were eaten by their mother after the first *GFP* screening, we failed to investigate their expression patterns in tissues.

### Amplification of transposon insertion sites

Ligation PCR was used to clone the transposon insertion sites as described previously (49). For zebrafish, stable transgenic lines were outcrossed with WT fish to generate positive offspring. Each line was outcrossed three times and at least three batches of embryos from different outcrossing were used for ligation PCR. For mice, the tails were used for genomic DNA extraction and used for ligation PCR. The transposon-specific and linker primer sequences used here were ZBnest1 and LinkerNest1 as described above for the primary PCR, and ZBnest3 and LinkerNest2 as described above for the secondary PCR. The PCR products were purified from an agarose gel, subcloned into the pJET1.2 vector and sequenced. Finally, the junction sequence of the insertion site was amplified and mapped to the genome (GRCm38/mm10 or GRCz11/danRer11) in UCSC browser.

## RESULTS

### *ZB* is the youngest *Tc1/mariner* transposon in zebrafish

**Figure 1.**
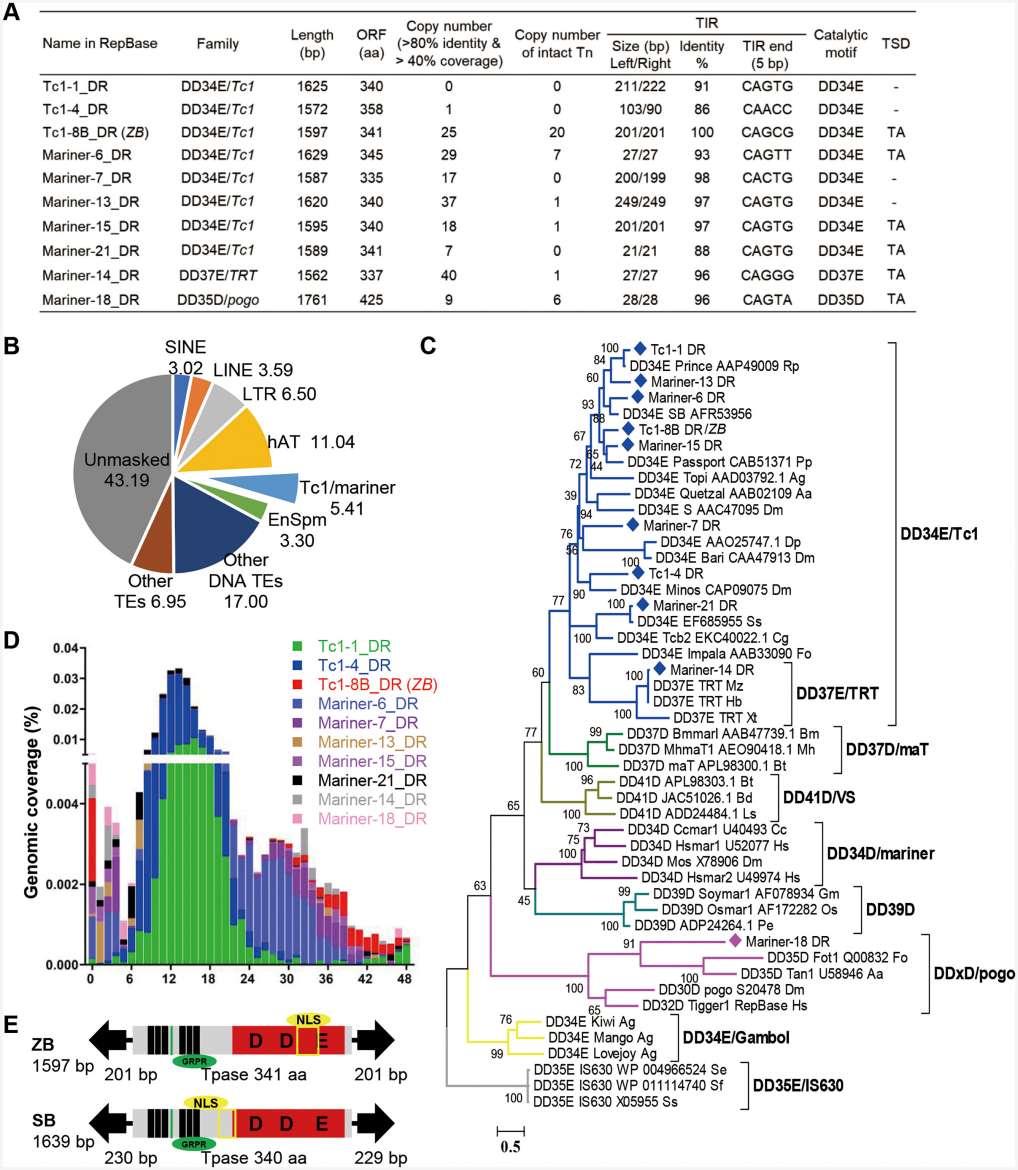
*Tc1/mariner* transposons in zebrafish. (**A**) Summary of the 10 autonomous transposons in the zebrafish genome. ORF, open reading frame; Tn, transposon; TIR, terminal inverted repeats; TSD, target site duplication. (**B**) Genomic coverage (5.41%) of *Tc1/mariner* transposons in the zebrafish genome. (**C**) Phylogenetic tree of the 10 transposons identified in this study with reference families of the *Tc1/mariner* transposons based on their transposases. Bootstrapped (1000 replicates) phylogenetic tree was inferred using the maximum likelihood method in IQ-TREE (37). Each sequence (with the exception of the DD39D subclasses) contained the name of the transposon, the gene sequence number corresponding to the transposon and the Latin abbreviation of the species in which the transposon was located. (**D**) Age distribution across the 10 *Tc1/mariner* subfamilies in zebrafish. The x-axis represents the insertion age (Mya, million years ago), and the y-axis represents the percentage of the genome composed of transposon families (%). (**E**) Transposon structure of *ZB* and *SB* transposon.

We sought and found 10 putative autonomous (Figure 1A) and 77 non-autonomous transposons (Supplementary Table S3) of the *Tc1/mariner* superfamily in the zebrafish genome via the joint annotation of mobilomes using a custom repeat library, which was constructed by multiple *de novo* approaches, as described in the Methods section. These analyses revealed that the *Tc1/mariner* superfamily is the second most abundant group of DNA transposons in the zebrafish genome and comprises 5.41% (90.69 Mb) of the zebrafish genome (Figure 1B). A phylogenetic tree based on the “DDE/D" domain sequences showed that eight of the autonomous *Tc1/mariner* transposons belonged to DD34E/*Tc1* families. One belonged to the DD37E/*TRT* family, which was also an intra-family of DD34E/*Tc1*; and one was classified as a member of the DD × D/*pogo* family (Figure 1C). The copy number of these autonomous transposons in the genomes of zebrafish varies dramatically, from zero to 40. Moreover, some of them (Tc1–8B_DR, Mariner-6_DR, Mariner-13_DR, Mariner-14_DR, Mariner-15_DR and Mariner-18_DR) contain intact copies, with very high sequence identities of left and right TIRs and are flanked by TA target site duplications (TSD) (Figure 1A), indicating that they may be functional elements. The autonomous transposons harbour a single open reading frame (ORF) encoding the full-length transposase flanked by TIRs, and all these transposases contain functional domains including a DNA binding domain (DBD), a catalytic motif (DDE/D), a nuclear localization signal (NLS) and a GRPR-like domain (Supplementary Figures S1 and S2), which are important for their transposition activity and indicate that these transposons may currently be active. An analysis of the insertion age revealed that the Tc1–8B_DR (previous name in RepBase) transposon (which was renamed as *ZB* due to it is a native transposon form in the ZeBrafish genome) was the youngest compared with the other autonomous *Tc1/mariner* transposons in the zebrafish genome, with almost all copies exhibiting an age distribution of less than 1 million years (Figure 1D).

*ZB* is highly similar to *SB* in structural organization, has a total length of 1597 bp and contains a single ORF predicted to encode a 341 amino acid (aa) transposase flanked by 201-bp TIRs (Figure 1E). Although the functional domains of transposases, including the DBD, DDE and GRPR motifs, are highly conserved between *ZB* and *SB*, the sequence identities of the left TIR, right TIR and transposases between *ZB* and *SB* are low, i.e. 40.1%, 46.4% and 51.3%, respectively (Supplementary Figure S3A, S3B). Furthermore, 20 full-length copies of *ZB* with >99% nucleotide identity to each other were identified in the zebrafish genome, 19 of which were intact and putatively functional copies (Figure 1A, Supplementary Figure S4). The TIRs and transposases of full-length *ZB* copies exhibited 100% and 99% sequence identity, respectively (Supplementary Table S4, Figure 1A, Supplementary Figure S4), suggesting again that *ZB* may be a relatively young component of the zebrafish genome, and may be potentially active.

### High transposition activity of *ZB* in mammalian cells

**Figure 2.**
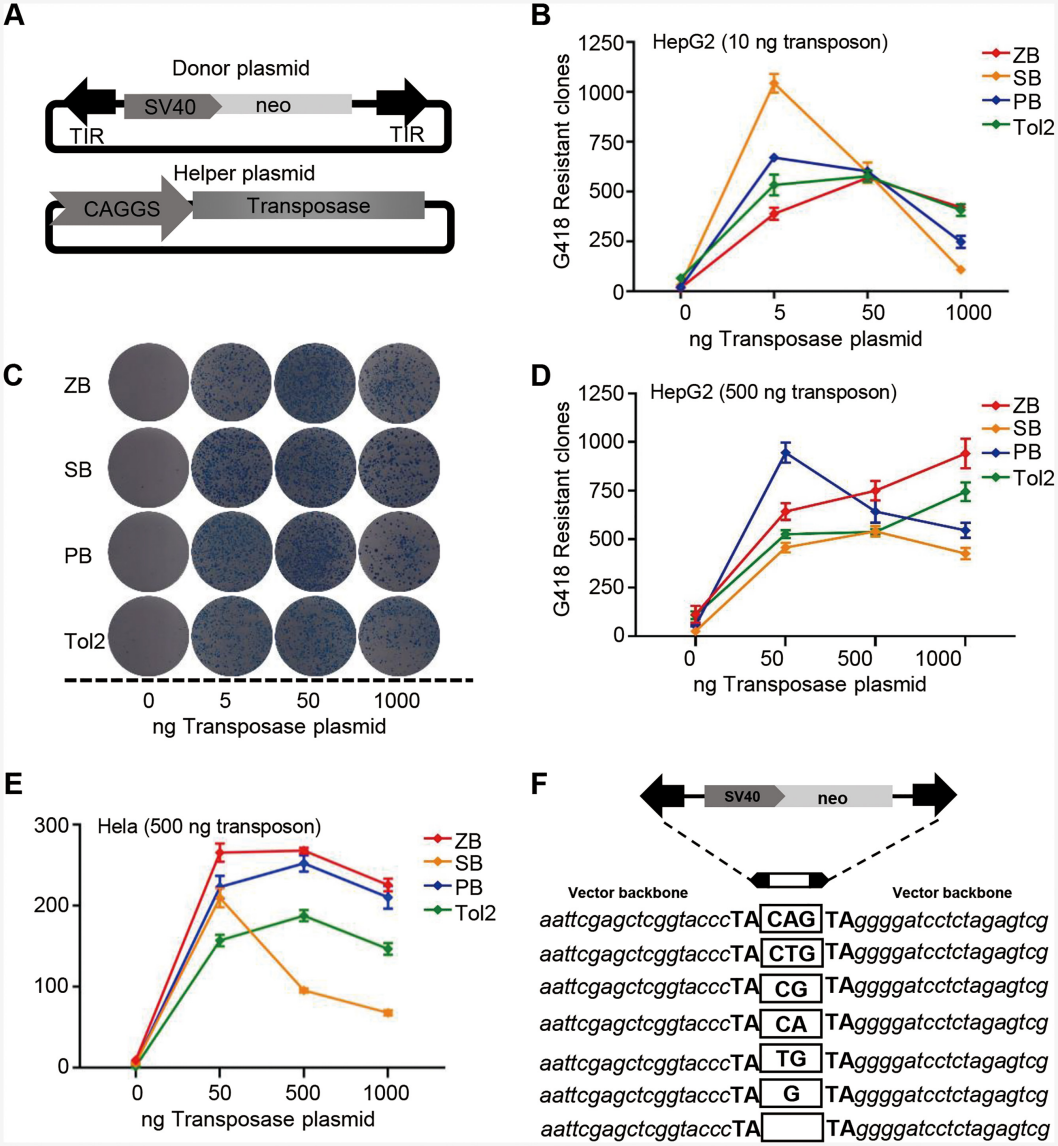
Transposition activities of *ZB*, *SB*, *PB* and *Tol2* in human cells. (**A**) Donor and helper plasmids used in human cells. Donor plasmids: the arrows represent transposon terminal inverted repeats (TIRs); *SV40*, *SV40* promoter; *neo*, neomycin resistance gene. Helper plasmids: *CAGGS*, *CAGGS* promoter; transposase, the transposase (*ZB*, *PB*, *SB*, or *Tol2*) coding gene. (**B**) Comparative transposition activities of *ZB*, *SB*, *PB* and *Tol2* in HepG2 cells co-transfected with low transposon DNA conditions (10 ng). (**C**) Stable colonies from HepG2 cells co-transfected with *ZB*, *SB*, *PB* and *Tol2* transposons in low transposon DNA conditions (10 ng). (**D**–**E**) Comparative transposition activities in HepG2 and HeLa cells co-transfected with the *ZB*, *SB100X*, *PB* and *Tol2* transposons in high transposon DNA conditions (500 ng). (**F**) Excision footprint of *ZB*. A schematic representation of the donor is shown on top. The pUC19 vector backbone sequences that flank the element in the donor construct are shown in italics. The transposon footprints are depicted in the white box.

A binary co-transfection assay system consisting of both a donor and a helper plasmid, which has been popularly used as a transposition test (30,50), was applied to validate *ZB* transposition activity and *ZB*-mediated chromosomal integration preference in cultured cells. The donor plasmid contained the *ZB* elements in which the *ZB* transposase (*ZB*ase) coding region was replaced by a drug-selection marker, while the helper plasmid carried the *ZB*ase ORF, but lacked the TIR sequences that are required for transposition. Furthermore, popular transposons, including *SB*, *PB* and *Tol2*, were also included for parallel comparison (42). To minimize the difference between systems and to ensure that only the transposon-specific TIR and transposase sequences are different between the vectors, all the transposon sequences containing an SV40-neo selection cassette were inserted at the same site in the donor plasmids (42), and the transposase-coding regions of *ZB*, *SB100X*, *PB* and *Tol2* were cloned into the same expression vectors (42) (Figure 2A).

To compare the transposition activities of the four transposons in an unbiased fashion and evaluate the putative overproduction inhibition (OPI) of *ZB*, a phenomenon that results in the inhibition of mobility activity by excess transposase expression (51), the amount of transposase helper plasmid for each transposon system was optimized using low (10 ng of DNA per 3 × 10^5^ transfected cells) and high (500 ng of DNA per 3 × 10^5^ transfected cells) dosages of transposon donor plasmids. First, we generated two independent transposition curves for the two different transposon dosages in transfected HepG2 cells. At the low dose of transposon donor plasmids, all transposons showed OPI, and *SB* and *PB* reached their peak activities at a transfection concentration as low as 5 ng of the transposase plasmid, while *ZB* and *Tol2* required higher amounts (50 ng) of transfected helper plasmid to obtain their maximal activities (Figure 2B, C). At the high amounts of transposon donor plasmids, *SB* and *PB* displayed typical OPI and reached their peak activity at 500 ng and 50 ng of transposase, respectively, while *ZB* and *Tol2* did not show OPI (Figure 2D, Supplementary Figure S5 and Table S5), which was inconsistent with the obvious OPI results of the *SB*, *PB* and *Tol2* transposons in HeLa cells (42), and may be attributed to cell differences. To confirm this hypothesis, OPI in the four transposons was also investigated in HeLa cells. The results demonstrated that all these transposon systems displayed obvious OPIs (Figure 2E, Supplementary Figure S6 and Table S6). In addition, *ZB* showed the highest transposition activity at 500 and 1000 ng of transposase and high dosages of transposon donor plasmids across the four transposons in both of HeLa and HepG2 cells (Figure 2D and E), indicating that *ZB* is a highly active transposon with great potential for application as a genetic tool. *Tc1/mariner* elements generate footprints in the range of 2−4 bp in the genome in the ‘cut-and-paste’ process (52). Here, we found that *ZB* transposition leaves footprints ranging from zero to three base pairs at the excision site (Figure 2F), similar to the footprints generated by *SB* and *Frog Prince* (33,53).

### Genomic insertion preferences of *ZB* in human HepG2 cells

**Figure 3.**
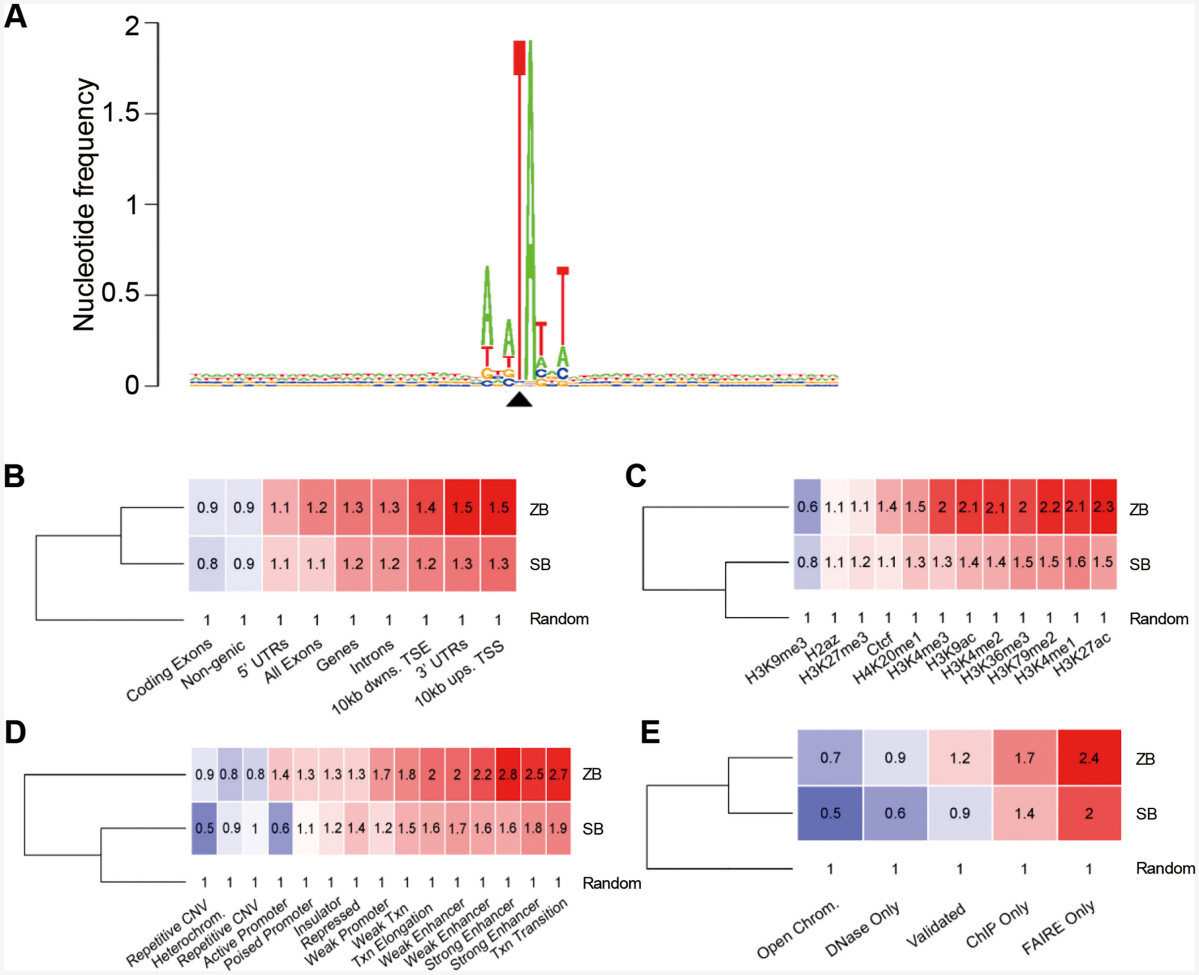
Distribution of *ZB* integration in the genome of HepG2 cells. (**A**) The sequence logo shows the consensus sequences at the genomic insertion loci in a 60 bp window around the target TA di-nucleotides. The value 2 (log2 4) on the y axis stands for maximum possible frequency. The black triangle indicates the position of the insertion site. (**B**) Comparison of *ZB* and *SB* insertion frequencies in gene-associated features of the human genome. The numbers indicate fold changes above the random expected frequency, set to 1. Insertion frequencies higher and lower than the random are color-coded in red and blue background, respectively. ‘Ups’: upstream, ‘dwns’: downstream, ‘TSS’: transcriptional start site, ‘TSE’: transcriptional end site. (**C**) Comparison of *ZB* and *SB* insertion frequencies in genomic segments with various histone modifications of the HepG2 genome. (**D**) Comparison of *ZB* and *SB* insertion frequencies in functional genomic segments. (**E**) Comparison of *ZB* and *SB* insertion frequencies in open chromatin measured by one or more of complementary methodologies (DNase-Seq, ChIP-Seq, FAIRE-Seq). The category ‘Open Chrom.’ was established by combining DNaseI-, and FAIRE-Seq results. ‘Validated’ stands for a dataset listing only regions that overlap between any methodologies. The dendrograms are based on the row means.

To assess the insertion preferences of the *ZB* transposon system, we performed a genome-wide insertion site profiling of *ZB* integrations in human HepG2 cells via high-throughput sequencing. We found that *ZB* prefers a palindromic AT repeat for insertion, with the six bases directly surrounding the insertion site forming a short, palindromic AT repeat (ATATATAT) in which the central TA is the actual insertion site (Figure 3A). This was very similar to that reported for the *SB* (54) and *Bari* (55) transposons, which could be common for *Tc1*-like elements. Subsequently, we compared the integration profile of *ZB* with that of *SB* in HepG2 cells using a computationally generated random integration dataset as a control. 198 048 independent *ZB* integration sites were obtained and analysed. Generally, the insertion preferences of *ZB* relative to the functional genomic regions were different from those of *SB* (Figure 3), with slight enrichments of *ZB* insertions in gene bodies, in genomic segments with histone marks associated with transcription and with activating gene regulatory regions and in open chromatin. These findings were in good agreement with those obtained with HepG2 specific functional genome segmentation data, which showed the most pronounced enrichments in enhancers and in loci around active transcriptional start sites. Therefore, *ZB* insertions display a more profound preference for transcription-promoting regulatory regions in the human genome than *SB*.

### *ZB* as a tool for enhancer trapping in zebrafish

**Figure 4.**
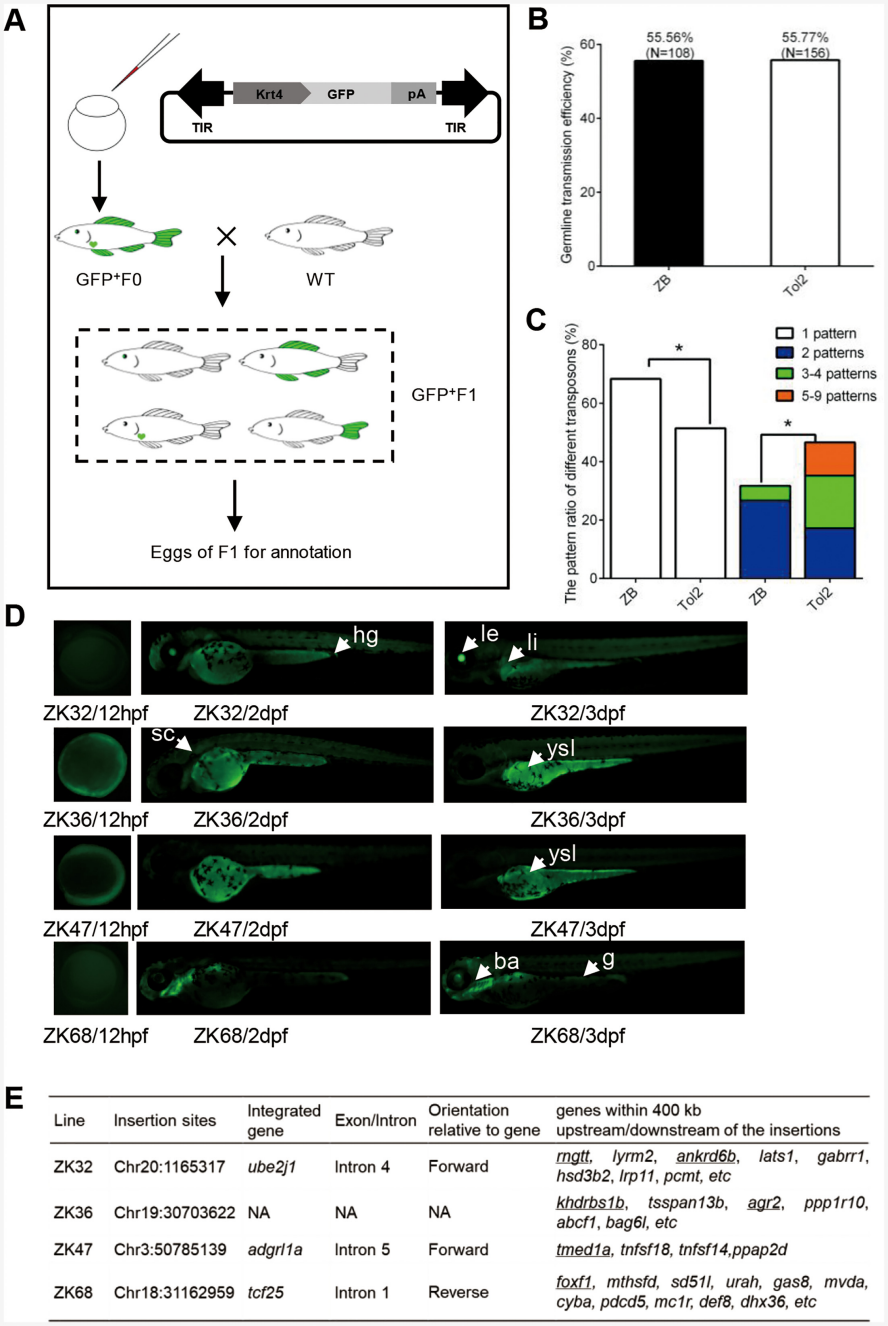
*ZB* transposon as a transgenic tool in zebrafish. (**A**) ET constructs used in zebrafish. The arrows represent transposon TIRs; *Krt4*, *Krt4* minimal promoter; *GFP*, reporter (green fluorescent protein) gene; pA, β-globin polyA. (**B**) Comparison of germline transmission efficiency mediated by the *ZB* and *Tol2* transposons by screening *GFP* expression in the F1 generation. (**C**) Different *GFP* patterns of F1 offspring generated from *ZB*- and *Tol2*-mediated transgenic zebrafish. (**D**) Fluorescence microscopy images of four ET lines from *ZB*-mediated transgenic zebrafish at 12 hpf, 2 dpf and 3 dpf. The arrows and arrowheads indicate distinct expression domains with resemblance to the activity of genes in the landing site environment. Abbreviations: ba, branchial arches; sc, spinal cord; g, gut; hg, hindgut; le, lens; li, liver; ysl, yolk syncytial layer. (**E**) Insertion sites of four ET lines. The predicted target genes that are expressed in similar domains to that shown by the ET lines are underlined.

The *ZB* transposon was also evaluated as a potential genetic tool for ET in zebrafish, which is an important vertebrate model. A *ZB* transposon-based ET vector (pZB-krt4-GFP) containing an ET box harbouring a mini-promoter (*Krt4*), a *GFP* reporter gene and a β-globin polyA, and flanked by *ZB* transposon TIRs (Figure 4A) was designed and constructed as described in the Materials and Methods section. The mini-promoter by itself has an extremely low basal level of activity and must be activated by a trapped enhancer to achieve detectable expression of a reporter gene. The *Tol2* transposon, which is popularly used in zebrafish transgenesis, was used as a control. The transgenic ET lines were generated by microinjection of the mixture of pZB-Krt4-GFP and *ZB* transposase mRNA, which were prepared as described in the Methods section. Embryos that showed *GFP* expression at 5 days post-fertilization (dpf) were raised for further germline transmission and *GFP* expression analysis. The embryos that survived to adulthood (F0) were separately outcrossed with WT fish, to generate F1 embryos for *GFP* screening and expression pattern analysis. The germline transfer efficiency of *ZB* transposon was 55.56% (*N*_F0_ = 108), which was similar to that of *Tol2* (55.77%, *N*_F0_ = 156) (Figure 4B and Supplementary Table S7). The distribution of the *GFP* expression pattern in F1 embryos across the two transposon groups was also compared. We found that the transgenic fish generated using the *ZB* transposon were most likely to produce offspring with a single expression pattern, while the transgenic fish generated using *Tol2* tended to produce offspring with a greater number of expression patterns (≥2) (this difference was significant; *P* < 0.05, χ^2^ test) (Figure 4C). Ten stable ET lines (F1) exhibiting distinct patterns of *GFP* expression (Figure 4D, Supplementary Figure S7) were outcrossed with WT fish to generate F2 offspring for *GFP* expression screening and insertion site annotation, respectively. Four of them (ZK32, ZK36, ZK47 and ZK68) were annotated successfully and mapped to the zebrafish genome (GRCz11), and they were confirmed as new insertions with different genomic coordinates from those of native *ZB* copies. All four F1 ET lines were confirmed to harbour a single insertion by linker polymerase chain reaction (PCR) assay using embryos from different outcrossing events (at least in triplicate), with three of them having been inserted into the introns of genes and one in an intergenic region (Figure 4E). *GFP* screening from birth to 5 dpf zebrafish embryos revealed that the *GFP* expression patterns of these ET lines were similar to those of several neighbouring endogenous genes (Figure 4D, E), which was determined by gene expression patterns deposited in zfin (56–59), and verified by whole mount *in situ* hybridisation (data not shown). The *GFP* signal of ZK32 was detected in the lens, liver and gut, which was most similar to the expression profiles of the neighbouring *ankrd6b* (lens, liver and gut) and *rngtt* (eye and gut) genes. ZK36 zebrafish embryos expressed *GFP* in the yolk syncytial layer and gut, which was similar to the expression profiles of two neighbouring genes (*khdrbs1b* and *agr2*) (56,57). In contrast, ZK47 displayed *GFP* expression in the yolk syncytial layer, which was similar to that observed for the endogenous gene (*tmed1a*) (58). Interestingly, ZK68 had specific *GFP* expression in the pharyngeal arches and gut, which are both highly similar to that of the neighbouring gene *foxf1* (59), while the gene at the landing site, *tcf25*, was expressed in adaxial cells, the lens, mesoderm, notochord, polster and somites, which suggests that it is unlikely to be regulated by the same set of trapped enhancers that act via the long-range regulation of *foxf1* and the transgene.

### *ZB* as a genetic tool for sperm mutagenesis in mice

**Figure 5.**
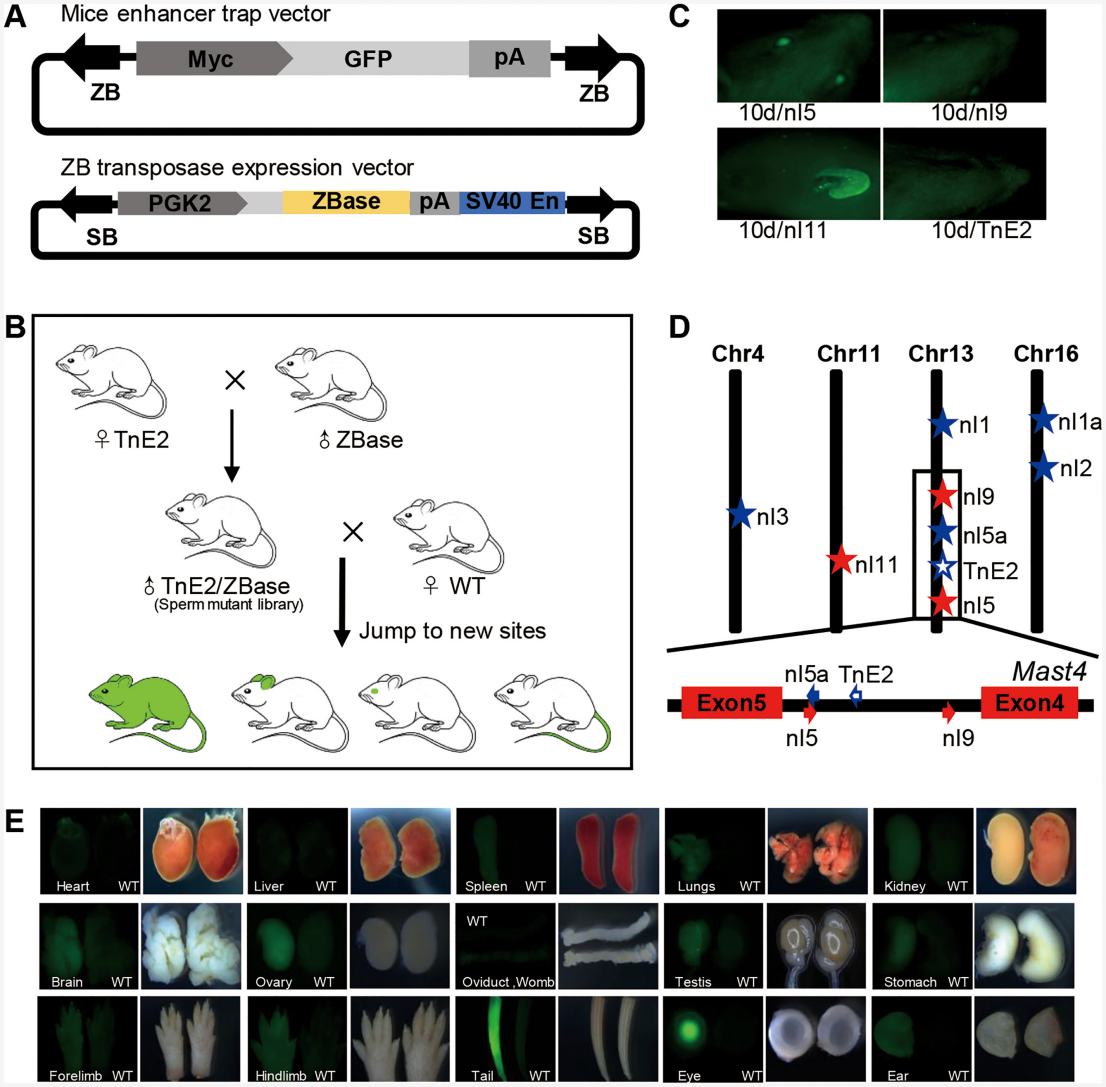
*ZB* transposon as a transgenic tool in mice. (**A**) ET constructs used in the mouse. The arrows represent TIRs; *Myc*, *Myc* minimal promoter; *GFP*, reporter (green fluorescent protein) gene; pA, Rabbit globin polyA. *ZB* transposase expression vector is mediated by *SB* transposon. *SB*, *SB* TIR; *PGK2*, human phosphoglycerate kinase 2 promoter; *SV40En*, *SV40* enhancer. (**B**) The procedure used for the generation of a sperm mutant library, which is a double-transgenic mouse containing both a transposon cassette and a transposase expression cassette. Mutant libraries can produce pups with new integration sites after mating with female WT mice. (**C**) Fluorescence screening in three pups (nl5, nl9 and nl11) revealed the presence of *GFP* positivity at 10 days of age, while the founder (TnE2) was *GFP* negative. (**D**) Diagram of transposon insertions in the mouse genome. The red stars represent GFP-positive insertions, the blue stars represent GFP-negative insertions and the hollow blue star represents the founder (TnE2). (**E**) *GFP* expression patterns of nl5 in multiple tissues and organs at 5 days of age.

We next explored enhancer trapping by the *ZB* transposon system *in vivo*, by directing transposition events in the male germline of the mouse, similar to the gene trapping systems previously established with the *SB* transposon in rats (60). Here, two transgenic mouse lines were developed to create a sperm mutation library. The first was a *ZB* transposase transgenic mouse, which expressed the *ZB* transposase specifically in germ cells in testis. To fulfil this requirement, we designed a vector (pSB-PGK2-ZBase) harbouring a *ZB* transposase expression cassette with a human phosphoglycerate kinase 2 (*PGK2*) promoter, to drive *ZB* transposase expression specifically in germ cells (44). The *ZB* transposase expression cassette was flanked by *SB* TIRs; thus, this vector (pSB-PGK2-ZBase) can be used for the generation of transgenic mice mediated by the *SB* transposon (Figure 5A). Twenty *ZB*ase transgenic mice, including five males, were generated efficiently (20/28 born, 71.4%), based on the microinjection protocol mediated by the *SB* transposon system, as described in the Methods section. The second was an ET transgenic mouse, which carried the ET vector mediated by the *ZB* transposon (pZB-Myc-GFP). The vector harboured an ET box, including a *GFP* reporter, a rabbit globin polyA and a human *Myc* minimal promoter, which has been proven to be sensitive to multiple mammalian enhancers (61), flanked by *ZB* TIRs (Figure 5A). Five ET transgenic mice (named as TnE1, TnE2, TnE3, TnE4 and TnE5), including two females and three males, were generated by microinjection of the *ZB* transposon (5/36 born, 13.9%). We chose TnE2, which contained an intact, single-copy *ZB* insertion and no *GFP* signal, as the founder mouse for the following crossing. Subsequently, the transposon (TnE2) and transposase (ZBase^+^) transgenic lines were selected for breeding to obtain males that carry both transgenes. These double-transgenic males were referred to as ‘sperm mutant library’ because they were the source of sperm carrying new *ZB* mobilisation events caused by ongoing transposition in the developing germ cells of these mice. Subsequently, four double-transgenic males (named as TnE2/Tp62, TnE2/Tp64, TnE2/Tp68 and TnE2/Tp191, the integration site annotation of which is summarized in Supplementary Table S8), were bred to WT females to generate offspring for ET screening. The general strategy used to carry out this procedure is shown in Figure 5B. In total, 135 pups from 12 litters were obtained and subjected to PCR analysis and *GFP* screening. Among these 135 pups, 53.3% (72/135) were PCR positive, 11 were the result of new mobilization events (Supplementary Figure S8A) and 61 were identical to the founder (Supplementary Figure S8B). The re-transposition rate (rate of generation of a new site or transposition) of the *ZB* (ET) system was 15.3% (11/72) (Table 1). Moreover, on average, one new insertion site was found per litter, which was higher than that reported for *SB* and *PB* (about 10%) in rats (60,62). Three pups (nl5, nl9 and nl11) were *GFP* positive at 10 days of age, as assessed using fluorescence screening. nl5 stemmed from a litter of 11 pups resulting from the seed mouse TnE2/Tp62, while nl9 and nl11 were from two litters of the TnE2/Tp64 seed mouse, with obvious *GFP* signals in the eyes and ears of nl5 and nl9, and in the ears of nl11 at this stage (Figure 5C and Table 1). The insertion sites of the 11 transgenic mice with re-transposition events were confirmed using linker PCR, as described in the Methods section, with eight of them mapping to the genome (the annotation results are summarized in Table 2). Four of them jumped into different chromosomes and four of them were reinserted into the same chromosomes, indicating that *ZB* also tended to exhibit local hopping, which has been reported for *SB* (63), *P* element (64), *Tol2* (65) and *Ac/Ds* (66). Interestingly, we found that the ET transgenes of nl5 and nl9 were reinserted into the same intron of the endogenous gene (*Mast4*), but with an opposite orientation to that present in the founder mice (TnE2), which did not show obvious *GFP* expression. This phenomenon was also observed in Ciona (67), in which the enhancers were sensitive to the orientation of the genes. A previous study showed that an ET vector inserted into the opposite direction to the endogenous gene can express the reporter gene (67). Although the mechanism underlying orientation sensitivity is not known, a plausible explanation is that the enhancer loses its access to promoters in an orientation-dependent manner (67). Moreover, the reinsertion of nl5 was remarkably close to the original integration site (<400 bp), while that of nl9 was located upstream (64 197 bp) of the original integration site (Figure 5D and Table 2). The ET transgene of nl11 was inserted into exon 2 of the endogenous gene (*Sost*) with opposite orientation (Figure 5D and Table 2). The nl5, nl9 and nl11 mice did not carry the *ZB* transposase transgene, and the ET transgene can be stable. *GFP* expression was detected in multiple organs of the nl5 mouse, including the spleen, lung, kidney, stomach, ovary, oviduct, womb, testis, eye, brain, ear, fore limb, hind limb and tail (Figure 5E); this was generally similar to the expression profile of the endogenous gene (*Mast4*), which exhibits ubiquitous expression as reported in NCBI.

**Table 1.** Sperm mutagenesis efficiency

Mutation library	No	PCR positive frequency^a^	New insertion frequency^b^	ET frequency^c^	ET transmission frequency^d^
TnE2/Tp62	1	4/8 = 0.5	1/4 = 0.25	-	-
	2	3/9 = 0.333	2/3 = 0.667	-	-
	3	5/11 = 0.455	1/5 = 0.2	1/11 = 0.091	4/8 = 0.5
TnE2/Tp191	1	11/15 = 0.733	1/11 = 0.091	-	-
	2	3/9 = 0.333	0/3 = 0	-	-
	3	10/15 = 0.667	1/10 = 0.1	-	-
TnE2/Tp68	1	9/13 = 0.692	1/9 = 0.111	-	-
	2	6/10 = 0.6	1/6 = 0.167	-	-
	3	7/13 = 0.538	0/7 = 0	-	
TnE2/Tp64	1	5/12 = 0.417	1/5 = 0.2	1/12 = 0.083	5/8 = 0.625
	2	6/13 = 0.462	1/6 = 0.167	1/13 = 0.077	7/11 = 0.636
	3	3/7 = 0.429	1/3 = 0.333	-	-
Total		72/135 = 0.533	11/72 = 0.153	3/135 = 0.022	-

^a^GFP-PCR positive per litter.

^b^New insertion of the GFP-PCR positive.

^c^visible GFP positive (F1) per litter.

^d^visible GFP positive (F2) per ET line litter.

No, number of litters.

**Table 2.** Detailed annotation of ET in mice

No	Line	Junction sequences	Insertion sites	Nearest gene	Intron or Exon	Orientation	Distance from origin site	GFP fluorescence
	TnE2	aggttccaggaaca**TA***cagcgggga*	Chr13:102860981	Mast4	4th intron	F	-	Negative
1	nl1	cctccacaggttca**TA***cagcgggga*	Chr13:55978551	Intergenic	N	N	46.9 Mb	Negative
2	nl1a	atactatctaccatgttata**TA***cagcgggga*	Chr16: 44756211	Nepro	1st exon	F	-	Negative
3	nl2	attttacatgtgta**TA***cagcgggga*	Chr16:76050830	Intergenic	N	N	-	Negative
4	nl3	gatgttacctattggctttc**TA***cagcgggga*	Chr4:67355475	Intergenic	N	N	-	Negative
5	nl5	aaaacgacagcaca**TA***cagcgggga*	Chr13:102860624	Mast4	4th intron	R	357 bp	**Positive**
6	nl5a	agtcacatggagtagc**TA***cagcgggga*	Chr13: 102860526	Mast4	4th intron	F	455 bp	Negative
7	nl9	ccagagtactgtga**TA***cagcgggga*	Chr13:102925178	Mast4	4th intron	R	64 197 bp	**Positive**
8	nl11	gcaagtgttataca**TA***cagcgggga*	Chr11:101962858	Sost	2nd exon	R	**-**	**Positive**

## DISCUSSION

In this study, we identified and characterized a highly active *Tc1/mariner* transposon (*ZB*) in the zebrafish genome, and evaluated its potential as a genetic tool for ET in zebrafish and in mice. Generally, *ZB* shared a similar structural organization and target site sequence preference, but had a slightly different integration profile as compared with the features of *SB* at the mammalian genome-wide scale (Figures 1 and 3). Furthermore, we provided strong evidence that *ZB* was a highly active DNA transposon in mammalian cells and that it could be used as an efficient transgenesis and mutagenesis tool in vertebrates (Figure 2). Although DNA transposons display great diversity in nature, and despite the fact that some of them are functional in their native form (68), only three of them (*SB*, *PB* and *Tol2*) have been well characterized and applied widely in vertebrate transgenesis and mutagenesis (8,69–71). *Minos* was also developed for transgenesis in vertebrates although it has not been widely used (72,73). Moreover, *SB* and *PB* also display great potential in human gene therapy (74,75). Both *PB* and *Tol2* were originally characterized as active transposons in native form (16,76), while the *SB* system was reconstructed by a computational phylogenetic approach (30). Both the *SB* and *PB* transposases have been optimized and improved significantly regarding their transposition activities, and exhibit great potential for genome engineering (77–80). Here, we identified a highly active *ZB* transposon in native form in the zebrafish genome based on bioinformatic analyses and cell-based functional tests. Both the age distribution and sequence identities of TIRs and transposase of *ZB* copies in the genome support the contention that *ZB* is a noticeably young invader in the zebrafish genome and is still putatively active in the zebrafish genome.

Within a certain concentration range more transposase in the cells results in an increase in transposition (81), while a transposase concentration exceeding an optimal concentration in a given cell leads to an inhibitory effect known as OPI, which has been described for the popularly used *SB*, *PB* and *Tol2* transposons (42). It has been suggested that OPI operates during assembly of the transpososome and arises from the multimeric state of the transposase, mediated by a competition for binding sites within the transposon TIRs (82). Here, we demonstrated that all four tested transposons, including *ZB*, showed this typical OPI phenomenon under conditions of low donor plasmid (10 ng) in HepG2 cells and at high donor plasmid (500 ng) in HeLa cells (Figure 2). However, *ZB* and *Tol2* did not exhibit this trend at high donor plasmid (500 ng) conditions in HepG2 cells (Figure 2), suggesting that OPI differences exist among these four transposons. These data indicate that the OPI of a DNA transposon may also be sensitive to cellular factors in addition to the dosage of the donor and helper plasmids.

The integration site preferences of particular elements, as an important biological property of DNA transposons, may depend on primary DNA sequence and chromatin structure and are substantially different, ranging from essentially random to selective at the genome-wide scale, which can greatly affect the utility of transposon vectors for different applications (8). Both the *Tol2* and *PB* transposons exhibit preferential integration near transcription start sites and transcriptional regulatory regions (42,83–86), which can be particularly advantageous for their application in ET. Here, we investigated the target site preferences and integration site bias of *ZB. ZB*, similar to the *SB* transposon (54), prefers AT-rich palindromic sequences for integration. However, unlike *SB*, the integrations of *ZB* are slightly biased towards enhancers, and open chromatin (Figure 3).

*ZB* was also evaluated regarding its potential for ET in zebrafish and mice. In zebrafish, the *Tol2* transposon system has been widely used to create gene trapping and ET lines, as well as protein trapping lines with reasonable efficiencies (22,87). The comparative study of ET efficiency across different transposons demonstrated that *Tol2* had the highest ET efficiency (55.56%) compared with *SB* (38.36%) and *PB* (32.65%) (43). Here, we found that *ZB* efficiently generated ET lines at almost the same rate as that reported for *Tol2* (about 56%), indicating the great potential of *ZB* for ET application in zebrafish. In addition, comparative ET in zebrafish with *ZB* and *Tol2* transposons indicated significantly higher frequency of single expression patterns suggestive of higher frequency of single-copy integration per genome can be generated by *ZB* (Figure 4). Single integration events are preferable for applications such as *cis*-regulatory element analysis by transgenesis and may simplify genetic analysis and interpretation of position effects. It is noted that, on rare occasions, transposition in a target locus may lead to cryptic splicing between splice sites of tan endogenous gene and sites in the transgene vector, thereby leading to translation of a functional reporter if the splicing maintains the reading frame of the reporter. Analysis of transcription start site of transgene reporter transcript can identify such spurious splicing mediated transcript fusion. In mice, we generated a sperm mutant library to evaluate the ET efficiency by mimicking the gene trapping strategy that has been well established in rats (60,62,88). In this protocol, by generating two lines of transgenic animals, i.e. one carrying the transposon and another expressing the transposase in germ cells, we were able to obtain double transgenic males in which re-transposition occurred in the germ cells. We found that the frequency of germline remobilization of single-copy transposons in the genome mediated by *ZB* was 15.3%. However, the remobilization frequency mediated by the *SB* transposon in mice was substantially lower than that (17% to 50%), as reported by Ruf et al. (89). This may be attributable to differences in the promoter used for transposase expression, and the actual transposase variant used in the experiments. In addition, we obtained three GFP-positive ET pups after screening a total of 135 pups from 12 litters, yielding a frequency of enhancer detection of 2.22% (3/135), which was dramatically lower than that (about 60%) observed in Ruf's study (89). The low efficiency of ET in our experiments may be attributed to the mini-promoter, which has proven to have a significant effect on ET trapping efficiency in zebrafish (21). The *Myc* mini-promoter from mice has been tested in mammalian cells (90). However, our data suggest that it may not be suitable for ET screening *in vivo*. Therefore, the optimization of a mini-promoter for ET in mice is highly recommended.

Irrespective of what method is used to deliver a reporter in ET applications, both the design of the reporter cassette as well as downstream analysis of interacting enhancers are important considerations. First, in general, the choice of promoter in the reporter cassette may influence ET efficiency (e.g. (91)), and future studies will be required to identify the sequence determinants in promoters, which define their interaction capacity with enhancers and inform on promoter choice for ET applications. Second, it remains a major challenge to identify the exact enhancer(s) which interact with the reporter cassette at the insertion site of the reporter, partly due to the potentially very large range of enhancer-promoter interactions. Recent 3D topology analysis tools such as CaptureC may allow detection of 3D interactions with multiple enhancers and reduce the potentially large number of candidates. In addition, advanced genomic technologies and genome-wide databases like the Encyclopedia of DNA Elements (ENCODE), or the functional annotation of the mammalian genome (FANTOM) project leading to enhancer atlases such as the Ensembl regulatory build may aid the identification and analysis of candidate enhancers recovered from ET screens.

In summary, the current study showed that *ZB* is a noticeably young invader of the zebrafish genome, and is highly active in zebrafish as well as mammalian cells. Our experiments provided the first step towards the establishment of a highly efficient mutagenesis tool in zebrafish and mice and suggest that the *ZB* system could be used as a powerful tool for genetic manipulations.

## DATA AVAILABILITY

The sequencing data generated from this study are available at NCBI: SRR13212054.

## Supplementary Material

gkab045_Supplemental_Files
