# Supplementary material for: A native, highly active Tc1/mariner transposon from zebrafish (ZB) offers an efficient genetic manipulation tool for vertebrates
Source: Nucleic Acids Res. 2021 Feb 8;49(4):2126–40. doi: 10.1093/nar/gkab045 (PMC7913693; doi:10.1093/nar/gkab045)
Supplement: gkab045_Supplemental_Files [file gkab045_supplemental_files.zip › Supplymental tables and figures.docx]

### Supplementary Table S1. Primers for plasmid construct, sequencing and PCRs.

| Primers | Sequence (5’-3’) |  |
| --- | --- | --- |
| **Plasmid construct** | |  |
| ZB_5IR.F | at*GGCGCGCC*TACAGCGGGGAAAATAAGTA |  |
| ZB_5IR.R | at*TGGCCAGCTAGCTCGCGA*TCATGTGTTCAATACTTATTC |  |
| ZB_3IR.F | at*TCGCGA*GTAAAGTGTGTTCAATACTTATT |  |
| ZB_3IR.R | at*GGCCGGCC*ATACAGCGGGGAAAATAAGTAT |  |
| ZB/XhoI/Kozak | *T*ACCA*CTCGA*GCCACCATGATGGGTAAAAACAAAGAACTC |  |
| ZB/NotI | AGCAACTA*GCGGCCGC*TTAATACTTTGTAGAAAAGCCTT |  |
| Myc/Spe1 | gc*ACTAGT*CTGCTACGGAGGAGCAGCAG |  |
| Myc/EcoR1 | at*GAATTC*TCGCTCCCTCTGCCTCTCGC |  |
| **Excision footprints** | | |
| puc1F | CCTCTGACACATGCAGCTCCCGG |  |
| puc1R | CAGTAAGAGAATTATGCAGTGCTGCC |  |
| puc2F | TCACAGCTTGTCTGTAAGCGG |  |
| puc2R | TCTTTCCTGCGTTATCCCCTGATTC |  |
| puc3F | TTCGCCATTCAGGCTGCGCAACTG |  |
| puc3R | CAGCTGGCACGACAGGTTTCCCG |  |
| **Illumina sequencing** | |  |
| ZBnest1 | GTGTCAGTAGCCCACTTAGAAATCCC |  |
| ZBnest2 | AATGATACGGCGACCACCGAGATCTACACTCTTTCCCTACACGACGCTCTTCCGATCTGTGTCAAATACTTATTTTCCCCGC |  |
| ZBnest3 | GTGTCAAATACTTATTTTCCCCGC |  |
| LinkerNest1 | GTAATACGACTCACTATAGGG |  |
| LinkerNest2 | GTCTCGTGGGCTCGGAGATGTGTATAAGAGACAG |  |
| **Integration sites** |  |  |
| GFP-F | CACCATGGTGAGCAAGGGCG |  |
| GFP-R | TTGTACAGCTCGTCCATGCC |  |
| GFP-JYZD | GACCACTACCAGCAGAACACC |  |
| TnE2-D | TAACGCACGCGAGGTAACAT |  |

### Supplementary Table S2. Sequences of *ZB* 5’ and 3’ TIR, and transposase TA clones.

| Name | Sequence (5’-3’) |
| --- | --- |
| ZB 5’TIR | cagcggggaaaataagtatttgacacatcagcatttttatcagtaaggggatttctaagtgggctactgacacaaaattcctaccagatgtagccatcaagccaaatattgaattcatacaaagaaatcagaacatttaagtatacaagttgagtcataataaataaagtgaaatgacacagggaataagtattgaacaca |
| ZB 3’TIR | tgtgttcaatacttattccctgtgtcatttcactttatttattatgactcaacttgtatacttaaatgttctgatttctttgtatgaattcaatatttggcttgatggctacatctggtaggaattttgtgtcagtagcccacttagaaatccccttactgataaaaatgctgatgtgtcaaatacttattttccccgctg |
| ZB transposase | atgatgggtaaaaacaaagaactctctcaagatctgcgtagtcttatcgttgaaaagcattttgatgggaatggttataggcgcatttccagaatgctgaatgttcctgtgagcactgtgggggctattatccggaaatggaaaaagcataagttcaccataaaccgaccacgatcaggtgctccacgtaagatccctgtccgaggagtccaaagaataatcaggagagttctccaagagccaaggaccactcgggcagaacttcaggaagacctcgcatcagcgggtactattgtttcaaagaaaactataagcaatgcactgaaccaccatggcatccatgcacgctcaccacgcaagactccattgctgaacaaaaagcatgttgaggcccggttaaagtttgcgaaacagcatttggagaagcctgtggattattgggaaactatagtatggtcagatgaaagcaaaattgaactttttggcagtcattctacacatcatgtttggagaagaaatggcactgcccaccaccccaagaacactataccaacagttaagtttgggggtggaagcatcatggtttggggctgcttttcagcaaggggtactggcagacttcatattattgaaggtaggatgaatggagaaatgtaccgggacattctggataaaaatctgctgccatctaccagaaagctgaaaatgaaaagagggtggacatttcagcaagacaatgatcccaaacacaaggccaaggaaacaatgaagtggtttcaaagaaagaaaatcaagttgcttgaatggcccagtcaatcacctgacctaaatcccatagaaaatctatggagagaactgaagatcaaagttcataaaagaggcccaaggaaccttcaagatttaaagaccgtttgtgtggaagaatgggccagaatcactcctgagcaatgcagacgactggtctctccatacaagaggcgtctagaagctgtgatcaccaacaaaggcttttctacaaagtattaa |

### Supplementary Table S3. The autonomous and non-autonomous transposons of Tc1/mariner families in zebrafish.

| No | Repbase ID: | Length (bp) | TIR | | | TSD |
| --- | --- | --- | --- | --- | --- | --- |
|  |  |  | Size(bp) | Identity% | TIR end  (5 bp) |  |
|  |  |  | Left/Right |  |  |  |
| 1 | Tc1-1_DR(Repbase) | 1625 | 211/222 | 91 | CAGTG | TA |
| 2 | Tc1-4_DR(Repbase) | 1572 | 103/90 | 86 | CAACC | TA |
| 3 | Tc1-8B_DR(Repbase) | 1597 | 201/201 | 100 | CAGCG | TA |
| 4 | Mariner-6_DR(Repbase) | 1629 | 27/27 | 93 | CAGTT | TA |
| 5 | Mariner-7_DR(Repbase) | 1587 | 199/200 | 98 | CACTG | TA |
| 6 | Mariner-13_DR(Repbase) | 1620 | 249/249 | 97 | CAGTG | TA |
| 7 | Mariner-15_DR(Repbase) | 1595 | 201/201 | 97 | CAGTG | TA |
| 8 | Mariner-21_DR(Repbase) | 1589 | 21/21 | 86 | CACTG | TA |
| 9 | Mariner-14_DR(Repbase) | 1562 | 27/27 | 96 | CAGGG | TA |
| 10 | Mariner-18_DR(Repbase) | 1761 | 28/28 | 96 | CCGTA | TA |
| 11 | Tc1-5_DR(Repbase) | 1234 | 26/26 | 100 | CAGGG | TA |
| 12 | Tc1-6_DR(Repbase) | 1203 | 170/170 | 100 | CAGGG | TA |
| 13 | Tc1-7_DR(Repbase) | 1646 | 90/90 | 94 | CAGTT | - |
| 14 | Tc1-8_DR(Repbase) | 1142 | 200/200 | 97 | CAGCG | - |
| 15 | TC1DR3B(Repbase) | 889 | 30/30 | 100 | CACTC | - |
| 16 | TDR1(Repbase) | 1236 | 52/52 | 90 | CAGTT | - |
| 17 | TDR2(Repbase) | 937 | 82/82 | 91 | CACTA | - |
| 18 | TZF28(Repbase) | 1609 | 216/220 | 72 | CAGTG | TA |
| 19 | TZF28B(Repbase) | 1614 | 217/217 | 99 | CAGTG | - |
| 20 | TZF28C(Repbase) | 1629 | 216/216 | 98 | CAGTG | - |
| 21 | Mariner-1_DR(Repbase) | 1508 | 169/154 | 83 | CAGGT | - |
| 22 | Mariner-2_DR(Repbase) | 1477 | 167/167 | 98 | CAGGT | TA |
| 23 | Mariner-3_DR(Repbase) | 2647 | 68/68 | 99 | CAGTT | - |
| 24 | Mariner-4_DR(Repbase) | 1627 | 270/267 | 98 | CAGTA | - |
| 25 | Mariner-5_DR(Repbase) | 1643 | 32/32 | 91 | CAGTC | TA |
| 26 | Mariner-8_DR(Repbase) | 1594 | 203/203 | 92 | CAGTG | - |
| 27 | Mariner-9_DR(Repbase) | 1613 | 230/230 | 98 | CAGTA | - |
| 28 | Mariner-10_DR(Repbase) | 1597 | 207/216 | 88 | CAGTC | - |
| 29 | Mariner-11_DR(Repbase) | 1347 | 59/60 | 93 | CACTC | - |
| 30 | Mariner-12_DR(Repbase) | 1614 | 221/224 | 82 | CAGTG | TA |
| 31 | Mariner-16_DR(Repbase) | 1249 | 23/23 | 96 | CACTG | - |
| 32 | Mariner-17_DR(Repbase) | 1600 | 66/67 | 87 | CAAAC | - |
| 33 | Mariner-19_DR(Repbase) | 1227 | 29/29 | 93 | CACTA | - |
| 34 | Mariner-20_DR(Repbase) | 1587 | 26/26 | 100 | CAGTT | - |
| 35 | DNA-TA-5_DR(Repbase) | 958 | 13/13 | 100 | CAGTG | - |
| 36 | Mariner-N1_DR(Repbase) | 270 | 70 | 96 | CACTC | - |
| 37 | Mariner-N2_DR(Repbase) | 3966 | 23/23 | 100 | CACTC | - |
| 38 | Mariner-N6_DR(Repbase) | 1652 | 29/29 | 90 | CAGTC | - |
| 39 | Mariner-N7_DR(Repbase) | 261 | 85/85 | 96 | CACTC | - |
| 40 | Mariner-N8_DR(Repbase) | 229 | 108/108 | 92 | CACTC | - |
| 41 | Mariner-N14_DR(Repbase) | 580 | 13/13 | 100 | CAGTG | - |
| 42 | Mariner-N15_DR(Repbase) | 1029 | 70/71 | 93 | CAGGG | - |
| 43 | Mariner-N17_DR(Repbase) | 901 | 85/84 | 92 | CAGTG | TA |
| 44 | Mariner-18N1_DR(Repbase) | 744 | 28/28 | 96 | CCGTA | - |
| 45 | Mariner-N19_DR(Repbase) | 1486 | 73/72 | 89 | CAGTT | TA |
| 46 | Mariner-N20_DR(Repbase) | 869 | 17/17 | 100 | CAGTA | TA |
| 47 | Mariner-N22_DRe(Repbase) | 1705 | 20/20 | 100 | CAGTT | TA |
| 48 | Mariner-N23_DRe(Repbase) | 1031 | 50/50 | 96 | CAGTT | TA |
| 49 | Mariner-N24_DR(Repbase) | 1357 | 56/56 | 100 | CAGTT | TA |
| 50 | Mariner-N25_DR(Repbase) | 933 | 19/19 | 89 | CAGTT | TA |
| 51 | Mariner-N26_DRe(Repbase) | 931 | 49/49 | 96 | CAGTT | TA |
| 52 | Mariner-N27_DR(Repbase) | 1186 | 24/24 | 88 | CAATT | - |
| 53 | Mariner-N28_DR(Repbase) | 1243 | 32/32 | 88 | CAGTT | TA |
| 54 | Mariner-N29_DR(Repbase) | 1765 | 68/68 | 94 | CAGTT | - |
| 55 | Mariner-N30_DR(Repbase) | 852 | 68/68 | 97 | CAGTT | - |
| 56 | Mariner-N31_DR(Repbase) | 540 | 28/28 | 100 | CACTA | - |
| 57 | Mariner-N32_DR(Repbase) | 228 | 27/27 | 100 | CAGTG | - |
| 58 | Mariner-N33_DR(Repbase) | 1156 | 33/33 | 94 | CAGTT | - |
| 59 | Mariner-N34_DR(Repbase) | 1112 | 26/26 | 100 | CAGTT | - |
| 60 | Mariner-N38_DR(Repbase) | 548 | 29/29 | 97 | CAGTT | - |
| 61 | Mariner-N42_DR(Repbase) | 1091 | 25/25 | 96 | CAGTT | - |
| 62 | Mariner-N45_DR(Repbase) | 1212 | 27/28 | 96 | CAGTT | - |
| 63 | Mariner-N46_Dr(new) | 211 | 36/36 | 100 | CAGGG | TA |
| 64 | Mariner-N47_Dr(new) | 189 | 88/88 | 90 | CAGGA | TA |
| 65 | Mariner-N48_Dr(new) | 230 | 26/26 | 100 | CAGTG | TA |
| 66 | Mariner-N49_Dr(new) | 328 | 29/29 | 100 | CAGTT | TA |
| 67 | Mariner-N50_Dr(new) | 346 | 31/31 | 94 | CAGTT | TA |
| 68 | Mariner-N51_Dr(new) | 392 | 29/29 | 100 | CAGTT | TA |
| 69 | Mariner-N52_Dr(new) | 481 | 29/29 | 100 | CAGTT | TA |
| 70 | Mariner-N53_Dr(new) | 546 | 28/28 | 100 | CAGTT | TA |
| 71 | Mariner-N54_Dr(new) | 917 | 22/22 | 86 | CAGTT | TA |
| 72 | Mariner-N55_Dr(new) | 1100 | 266/275 | 97 | CAGGG | TA |
| 73 | Mariner-N56_Dr(new) | 1028 | 85/85 | 91 | CAGTT | TA |
| 74 | Mariner-N57_Dr(new) | 1033 | 138/138 | 99 | CAGTG | TA |
| 75 | Mariner-N58_Dr(new) | 1201 | 458/458 | 100 | CAGGG | TA |
| 76 | Mariner-N59_Dr(new) | 1504 | 162/162 | 99 | CAGCC | TA |
| 77 | Mariner-N60_Dr(new) | 259 | 120/120 | 92 | CACTC | TA |
| 78 | Mariner-N61_Dr(new) | 257 | 117/117 | 92 | CACTC | TA |
| 79 | Mariner-N62_Dr(new) | 452 | 84/84 | 98 | CACTC | TA |
| 80 | Mariner-N63_Dr(new) | 503 | 82/82 | 91 | CACTA | TA |
| 81 | Mariner-N64_Dr(new) | 753 | 82/82 | 91 | CACTA | TA |
| 82 | Mariner-N65_Dr(new) | 934 | 82/82 | 91 | CACTA | TA |
| 83 | Mariner-N66_Dr(new) | 1110 | 30/30 | 100 | CACTC | TA |
| 84 | Mariner-N67_Dr(new) | 1147 | 30/30 | 100 | CACTC | TA |
| 85 | Mariner-N68_Dr(new) | 219 | 100/100 | 91 | CGCGA | TA |
| 86 | Mariner-N69_Dr(new) | 251 | 116/116 | 92 | CTCAG | TA |
| 87 | Mariner-N70_Dr(new) | 881 | 17/17 | 94 | CCAGG | TA |

Note: TIR, terminal inverted repeats; TSD, target site duplication. “-” represents not detectable.

### Supplementary Table S4. Twenty full-length copies of *ZB* in zebrafish genome.

| ID | Length  (Tn, bp) | Length  (Tpase, aa) | Chr. | Location | TSD |
| --- | --- | --- | --- | --- | --- |
| ZB_copy1 | 1597 | 341 | 8 | NC_007119.7:44207847-44209443 | TA |
| ZB_copy2 | 1597 | 341 | 17 | NC_007128.7:33958902-33960498 | TA |
| ZB_copy3 | 1597 | 341 | 18 | NC_007129.7:31141292-31142888 | TA |
| ZB_copy4 | 1597 | 341 | 21 | NC_007132.7:3634261-3635857 | TA |
| ZB_copy5 | 1597 | 341 | 10 | NW_018395287.1:190592-192188 | TA |
| ZB_copy6 | 1597 | 341 | 20 | NC_007131.7:17278631-17280227 | TA |
| ZB_copy7 | 1597 | 341 | 5 | NC_007116.7:1716494-1718090 | TA |
| ZB_copy8 | 1597 | 341 | 5 | NC_007116.7:60093584-60095180 | TA |
| ZB_copy9 | 1597 | 341 | 9 | NC_007120.7:52265193-52266789 | TA |
| ZB_copy10 | 1597 | 341 | 5 | NW_018394613.1:183016-184612 | TA |
| ZB_copy11 | 1597 | 341 | 1 | NC_007112.7:7987819-7989415 | TA |
| ZB_copy12 | 1597 | 341 | 4 | NC_007115.7:61995639-61997235 | TA |
| ZB_copy13 | 1597 | 341 | 4 | NC_007115.7:52993659-52995255 | TA |
| ZB_copy14 | 1597 | 341 | 19 | NC_007130.7:30289338-30290934 | TA |
| ZB_copy15 | 1597 | 341 | 24 | NC_007135.7:15669649-15671245 | TA |
| ZB_copy16 | 1597 | 341 | 2 | NW_018394510.1:430-2026 | TA |
| ZB_copy17 | 1597 | 341 | 23 | NC_007134.7:44806658-44808254 | TA |
| ZB_copy18 | 1597 | 341 | 23 | NW_018395163.1:32622-34218 | TA |
| ZB_copy19 | 1597 | 341 | 3 | NC_007114.7:36170736-36172332 | TA |
| ZB_copy20 | 1597 | 341 | 14 | NC_007125.7:14051630-14053226 | TA |

Note: Tn, transposon; Tpase, transposase; Chr, chromatin; TSD, target site duplication.

### Supplementary Table S5. Colonies from HepG2 cells co-transfected with *ZB*, *SB*, *PB* and *Tol2* transposons in high and low transposon DNA conditions (500 and 10 ng).

| Tn:Tpase  (ng) | ZB | | | SB | | | PB | | | Tol2 | | |
| --- | --- | --- | --- | --- | --- | --- | --- | --- | --- | --- | --- | --- |
| High dose |  |  |  |  |  |  |  |  |  |  |  |  |
| 500:0 | 195 | 50 | 92 | 7 | 33 | 37 | 49 | 60 | 53 | 128 | 127 | 68 |
| 500:50 | 558 | 696 | 672 | 419 | 448 | 502 | 946 | 894 | 997 | 546 | 506 | 526 |
| 500:500 | 799 | 699 | 749 | 487 | 571 | 563 | 560 | 613 | 753 | 527 | 518 | 565 |
| 500:1000 | 863 | 868 | 1093 | 373 | 430 | 473 | 485 | 535 | 617 | 744 | 792 | 696 |
| Low dose |  |  |  |  |  |  |  |  |  |  |  |  |
| 10:0 | 5 | 16 | 29 | 21 | 31 | 44 | 14 | 36 | 13 | 34 | 81 | 83 |
| 10:5 | 342 | 447 | 379 | 1044 | 963 | 1126 | 672 | 640 | 703 | 434 | 559 | 608 |
| 10:50 | 536 | 603 | 570 | 545 | 543 | 699 | 603 | 604 | 602 | 536 | 565 | 633 |
| 10:1000 | 440 | 413 | 412 | 89 | 128 | 110 | 253 | 299 | 194 | 437 | 438 | 349 |

### Supplementary Table S6. Colonies from Hela cells co-transfected with *ZB*, *SB*, *PB* and *Tol2* transposons in high transposon DNA conditions (500 ng).

| Tn:Tpase  (ng) | ZB | | | SB | | | PB | | | Tol2 | | |
| --- | --- | --- | --- | --- | --- | --- | --- | --- | --- | --- | --- | --- |
| 500:0 | 11 | 12 | 4 | 8 | 6 | 2 | 7 | 4 | 5 | 1 | 3 | 11 |
| 500:50 | 246 | 285 | 266 | 191 | 207 | 231 | 246 | 198 | 225 | 143 | 166 | 246 |
| 500:500 | 275 | 265 | 264 | 95 | 100 | 91 | 234 | 254 | 269 | 193 | 196 | 275 |
| 500:1000 | 237 | 229 | 211 | 74 | 65 | 64 | 193 | 238 | 200 | 133 | 158 | 237 |

### Supplementary Table S7. Screen summary of enhancer trapping in zebrafish.

|  | ZB | Tol2 |
| --- | --- | --- |
| Embryos injected | 451 | 487 |
| Positive embryos (GFP+) at 1 days | 324 | 435 |
| Positive embryos (GFP+) at 5 days | 248 | 288 |
| Number of F0 fish generating offspring | 108 | 156 |
| Number of F0 fish generating GFP+ offspring | 60 | 87 |
| Enhancer trapping efficiency (%) | 55.56 | 55.77 |

### Supplementary Table S8. Integration sites of ZBase mediated by *SB* transposon in mice.

| No. | Junction sequences | Insertion sites | Integrated  gene | Exon/Intron | Orientation |
| --- | --- | --- | --- | --- | --- |
| Tp62 | ctacacaaacacacaca**TA***cagttgaagt* | Chr2: 148447324 | intergenic | NA | NA |
| Tp64 | gttaacaataatcgtatt**TA***cagttgaagt* | Chr6: 94551216 | Slc25a26 | Intron 8 | Forward |
| Tp68 | gtcagctctccaagtta**TA***cagttgaagt* | Chr15: 35236470 | intergenic | NA | NA |
| Tp191 | tttcagatgaaagatta**TA***cagttgaagt* | Chr9: 152261203 | intergenic | NA | NA |

Note: NA represents not analyzed.


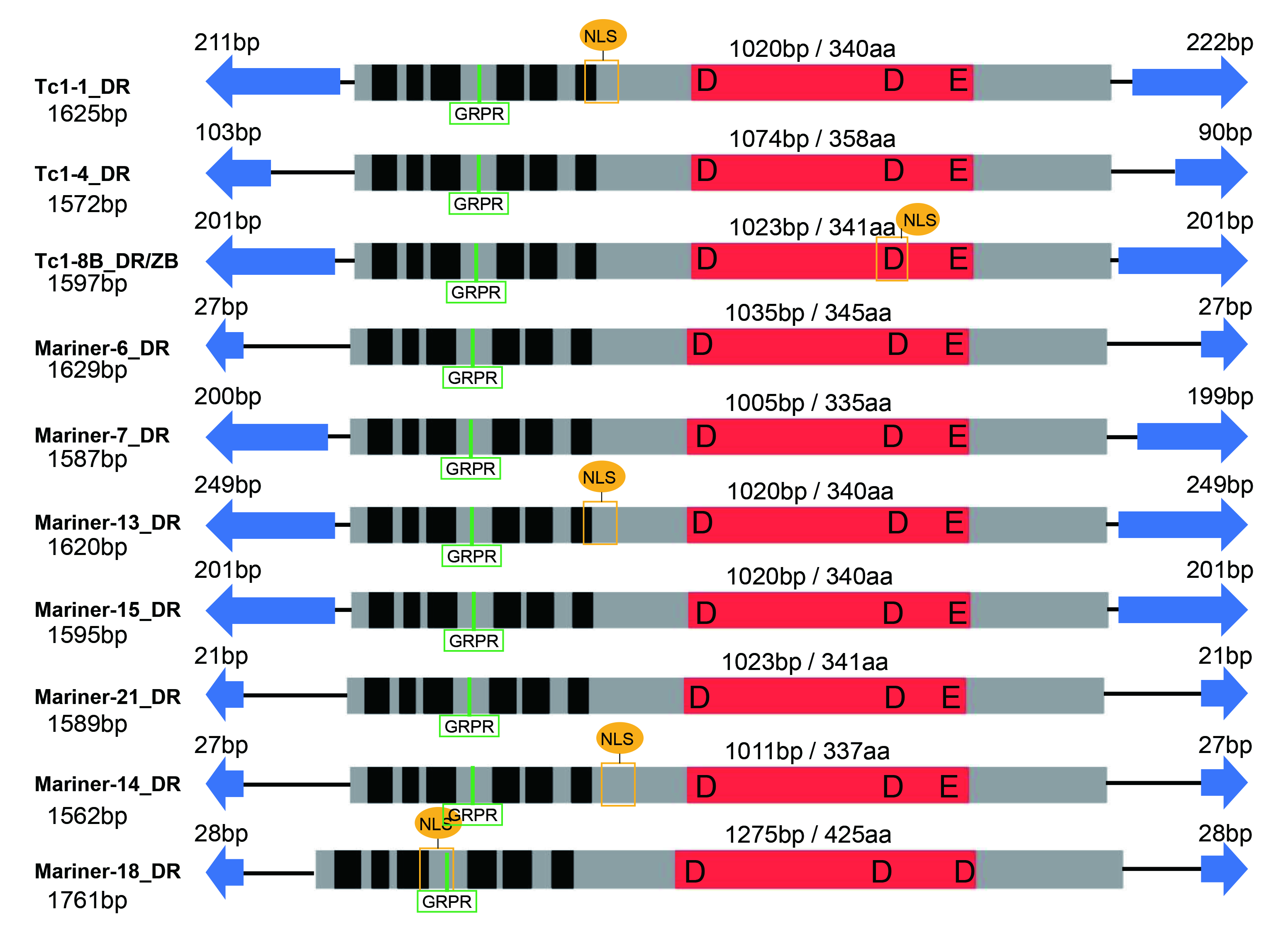


**Supplemental figures S1. Structural organization of *Tc1*/*mariner* transposons in zebrafish.** Structures of 10 *Tc1*/*mariner* transposons containing intact transposons. The blue arrows represent TIRs, the grey boxes represent transposase domains, the black bars represent helices, the red boxes represent catalytic domains and the numbers represent the length of TIRs and the position of residues.


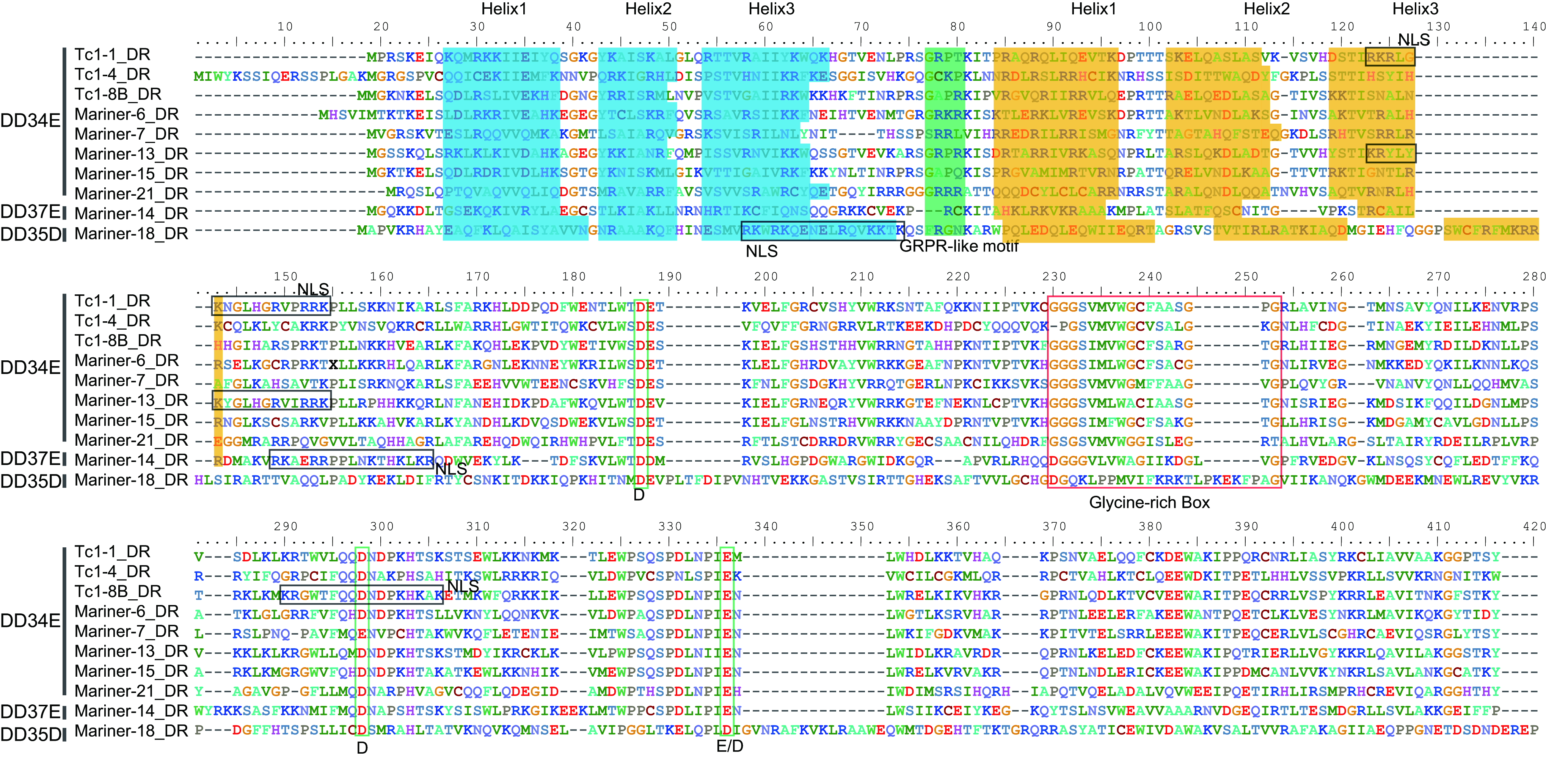


**Supplemental figures S2. Alignment of *Tc1*/*mariner* transposases in the zebrafish genome.** Eight transposons contained a DD34E catalytic domain, while the remaining two contained DD37E and DD35D domains, respectively. The first three helices are highlighted in blue; the second helices are high lightened in yellow; GRPR-motifs are shown in green; nuclear localization signals (NLSs) are indicated by a black box; and glycine-rich motifs are shown as a red box.


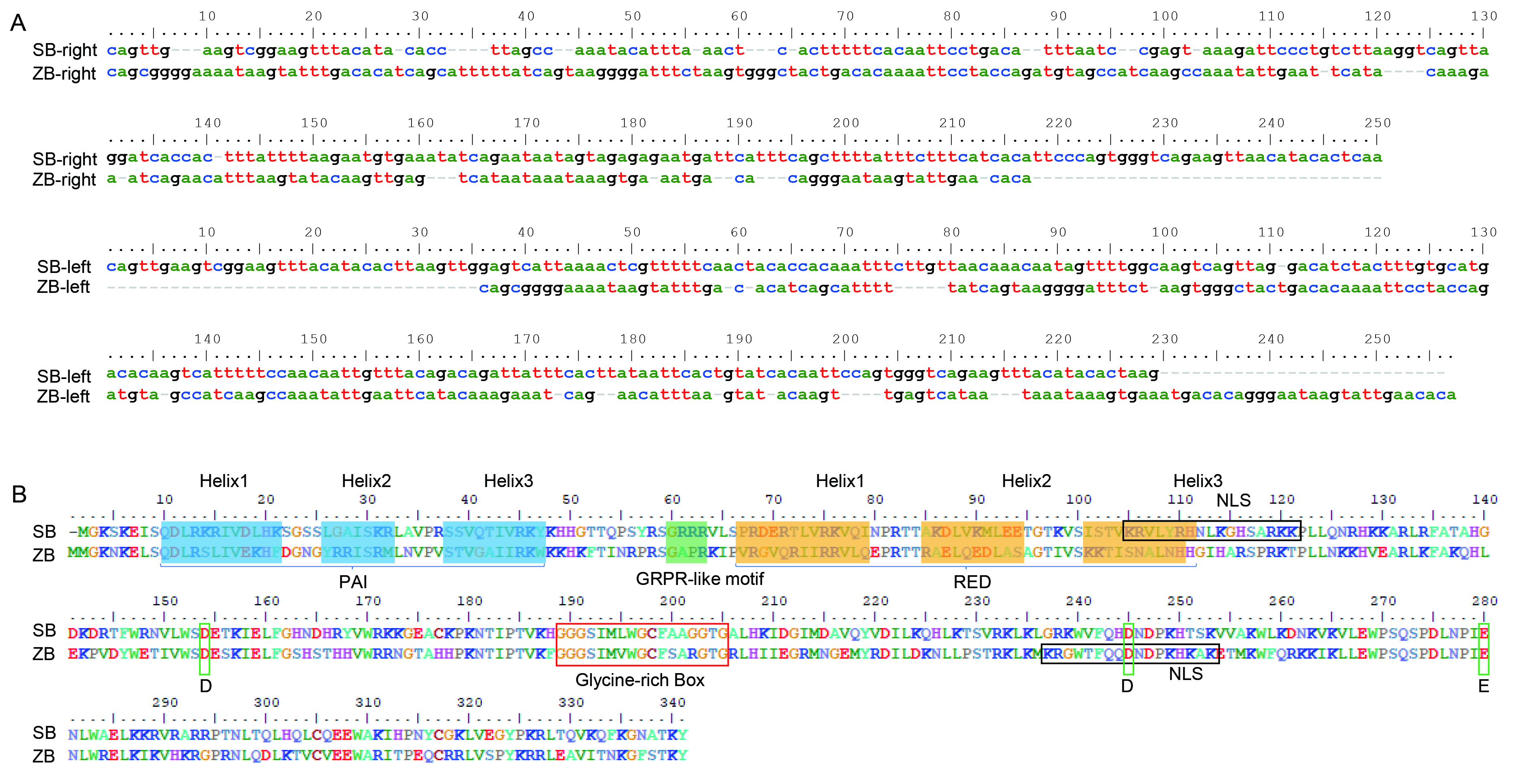


**Supplemental figures S3. Alignment of the TIRs and transposase of *ZB* and *SB*.** (A) Alignment of the terminal inverted repeats (TIRs) of *ZB* and *SB*. The identity of the *ZB* and *SB* terminal repeats was low (right, 46.4%; left, 40.1%). (B) Alignment of the transposases of *ZB* and *SB* (their identity was 51.3%). The first three helices are highlighted in blue; the second helices are highlighted in yellow; GRPR-motifs are shown in green; nuclear localization signals (NLSs) are provided in the black box; glycine-rich motifs are shown in the red box. Both graphs were produced by Bioedit graphic view.


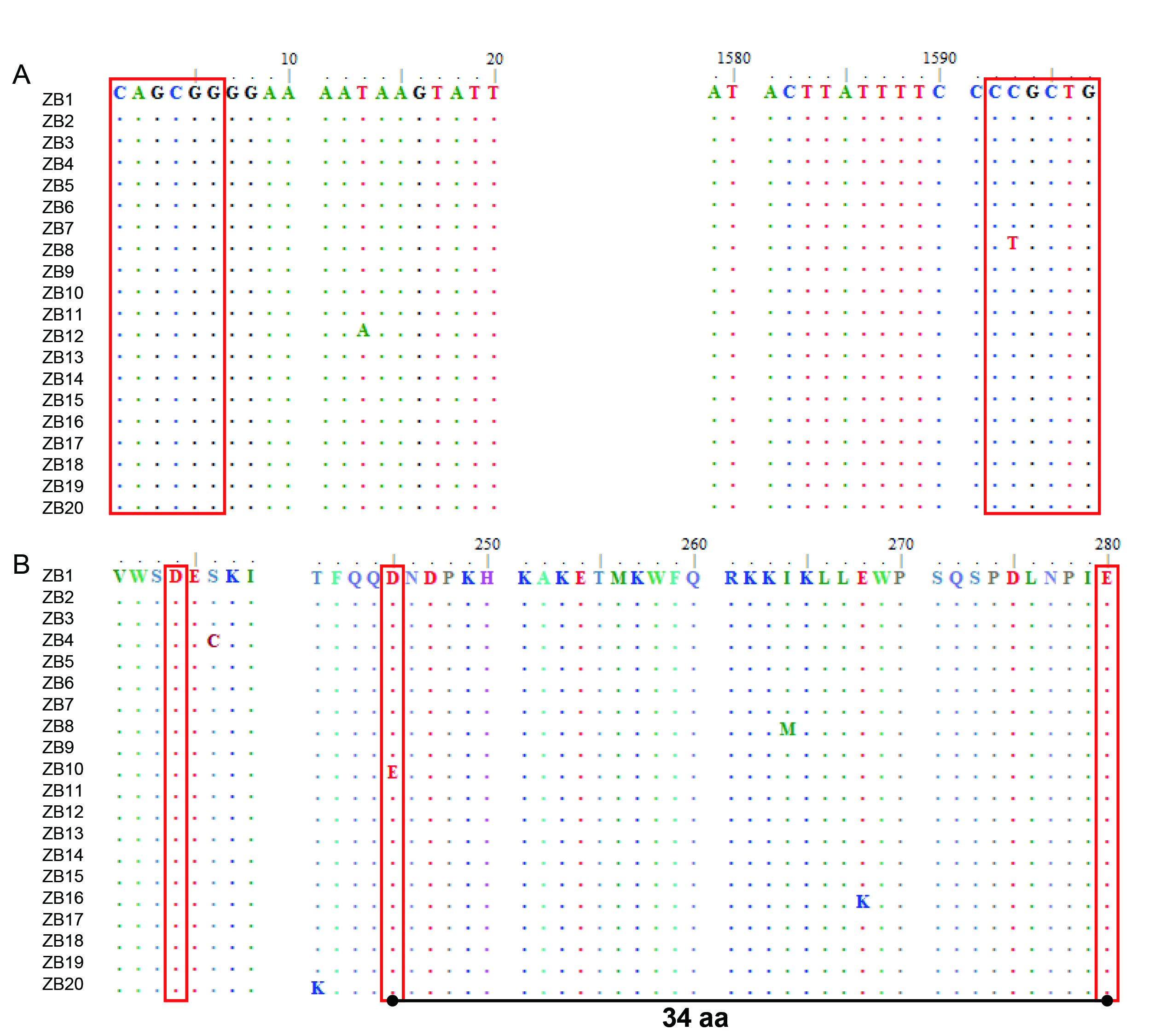


**Supplemental figures S4. Alignment of the TIR and transposase of *ZB* in the zebrafish genome.** (A) Alignment of *ZB* TIRs in the zebrafish genome. The last five base pairs are shown in red boxes. (B) Alignment of *ZB* transposases in the zebrafish genome. The three conserved amino acids, DDE, are shown in blue boxes. The number ‘34 aa’ represents the distance between the second D and E.


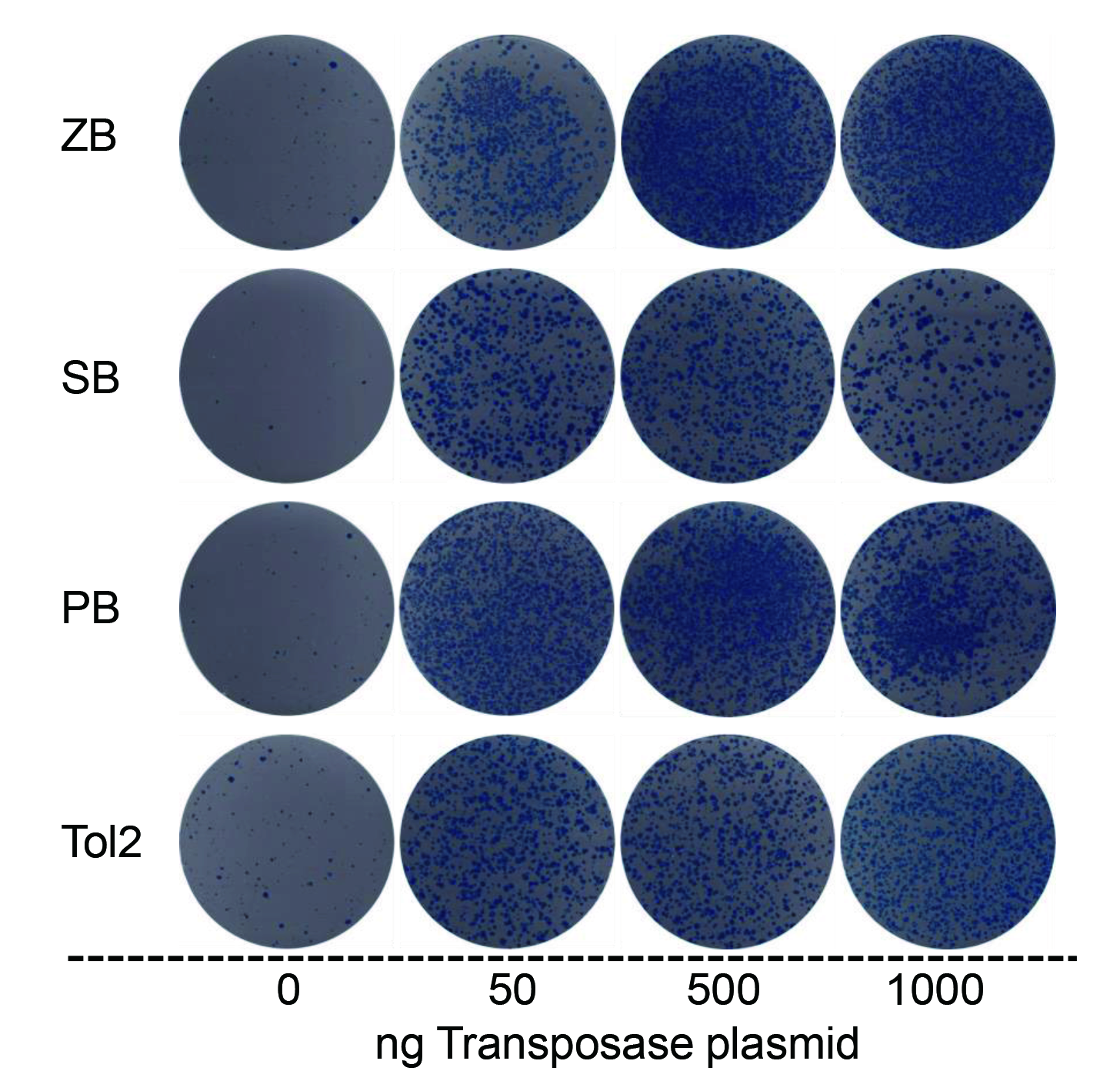


**Supplemental figures S5.** **Stable** **colonies from HepG2 cells co-transfected with *ZB*, *SB100X*, *PB* and *Tol2* transposons in high transposon DNA conditions (500 ng).** Transposition activity was measured under a fixed amount of transposon plasmid (500 ng) co-transfected with increasing amounts of transposase expression plasmids into HepG2 Cells. The total amount of transfected DNA was adjusted in each sample to 1.5 μg using pTNT. Stable colonies were stained with methylene blue.


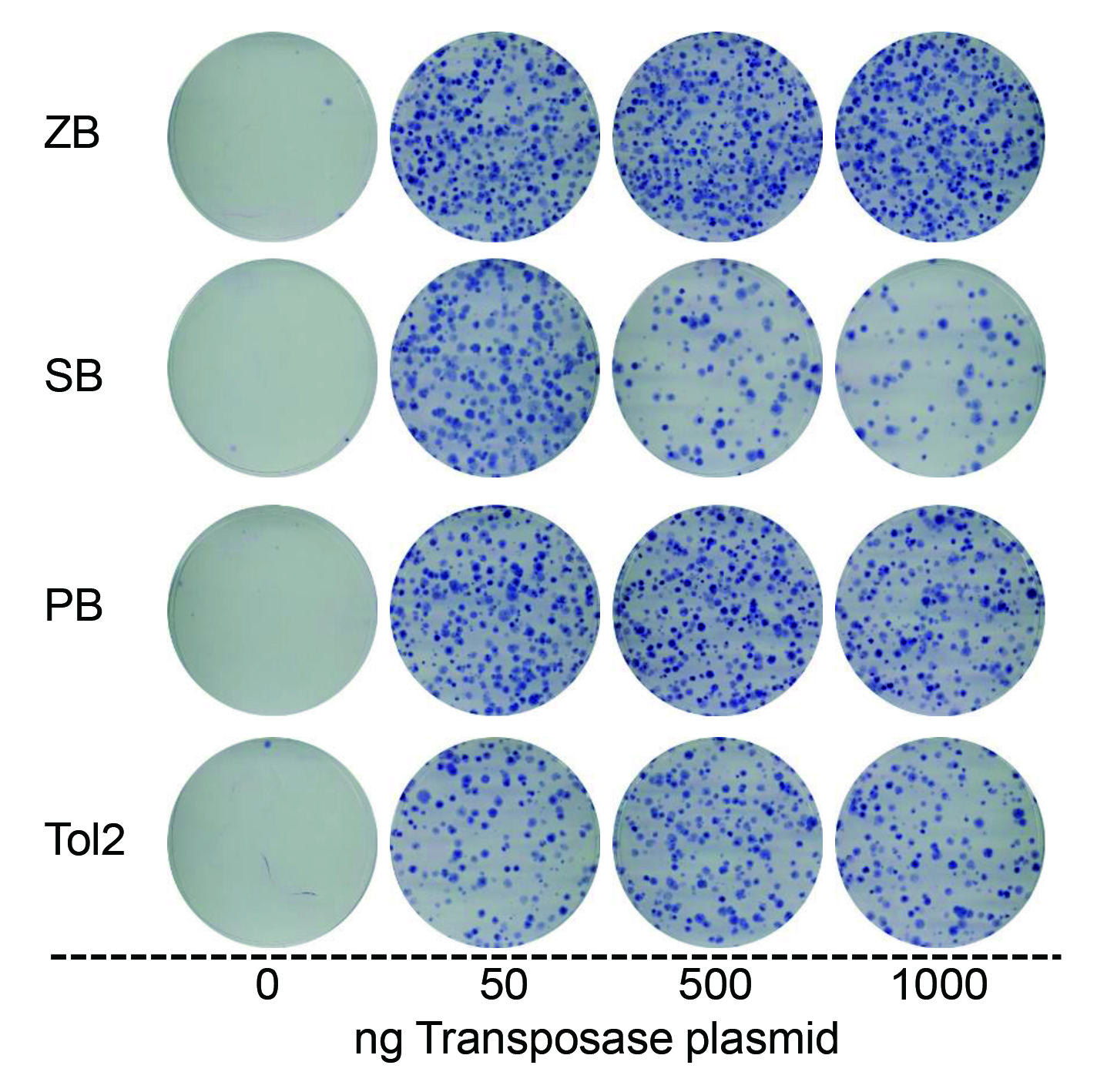


**Supplemental figures S6. Colonies from HeLa cells co-transfected with *ZB* transposon plasmids and 500 ng donor plasmids.** Transposition activity was measured under a fixed amount of transposon plasmid (500 ng) co-transfected with increasing amounts of transposase expression plasmids into HeLa Cells. The total amount of transfected DNA was adjusted in each sample to 1.5 μg with pTNT. Stable colonies were stained with methylene blue.


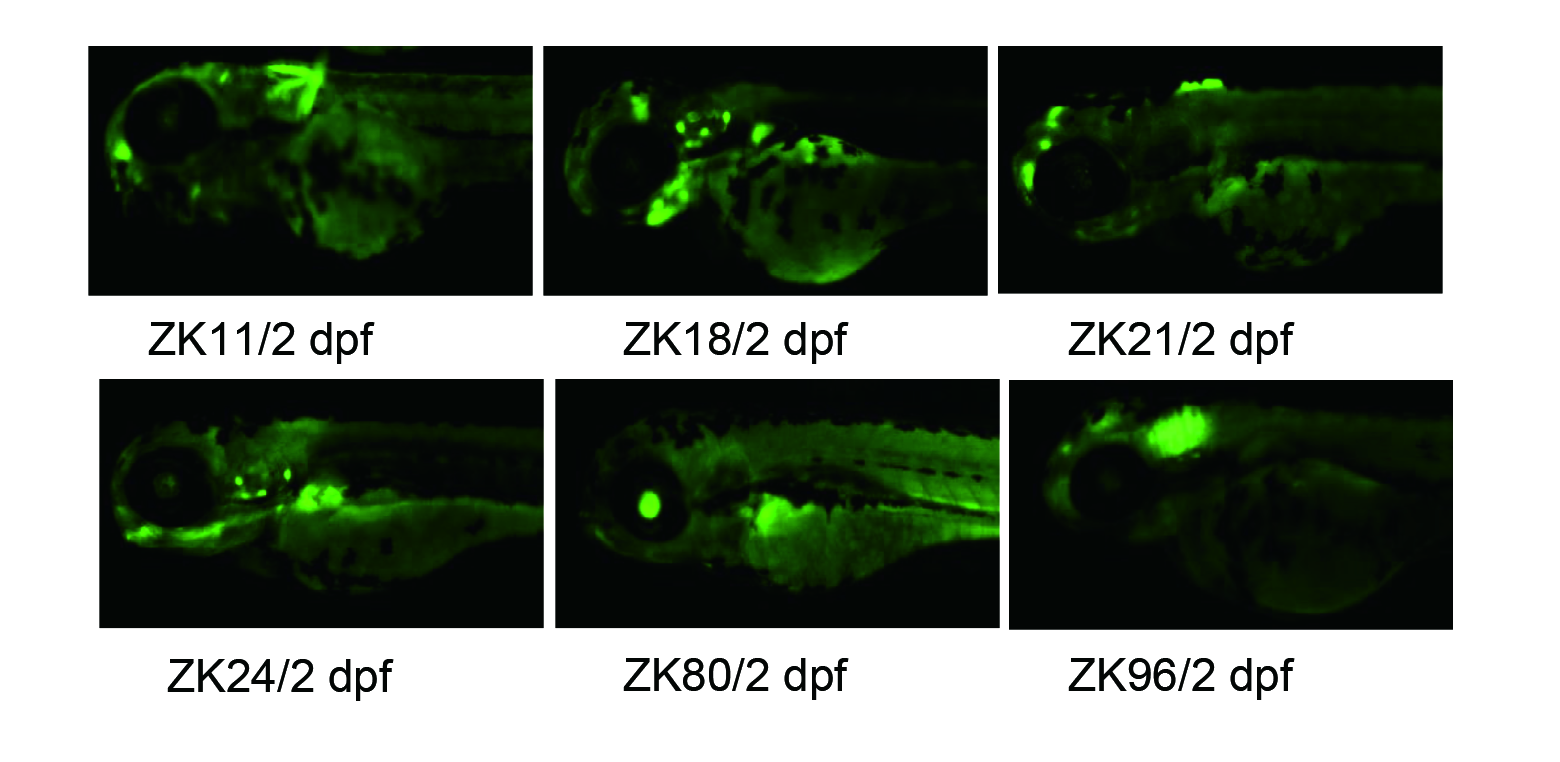


**Supplemental figures S7. Screening of GFP patterns of ET F1 offspring at 2 dpf.**


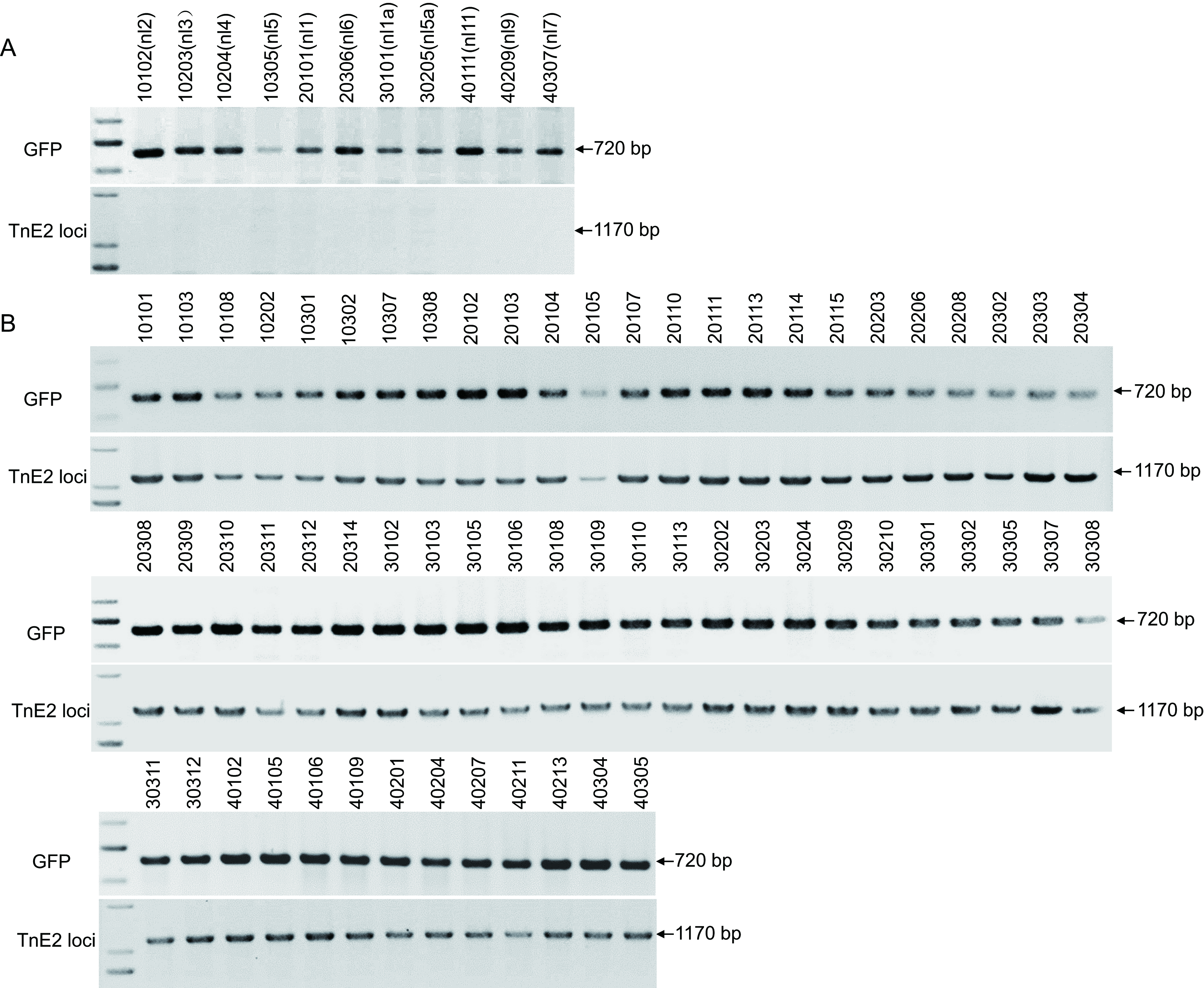


**Supplemental figures S8. PCR amplification of GFP and genomic flanked sequence.** All offspring were firstly screened by PCR amplification with GFP specific primers, then followed by PCR amplification with the TnE2 locus specific primers, the GFP positive, but TnE2 locus negative animals (eleven) were designated as new jumping events, and summarized in the Figure S8A, and animals (sixty-one) with both GFP and TnE2 locus positive were designated as original integrations and summarized in Figure S8B, the putative new jumping events (more than one insertion copies) were further excluded by Ligation mediated PCR for these animals.
